# Cognitive and Motivational Factors Associated with Sedentary Behavior: A Systematic Review

**DOI:** 10.3934/publichealth.2016.4.956

**Published:** 2016-11-28

**Authors:** Scott Rollo, Anca Gaston, Harry Prapavessis

**Affiliations:** Exercise and Health Psychology Laboratory, School of Kinesiology, Western University, London, Ontario, Canada

**Keywords:** sedentary behavior, psychological determinants, cognitive factors, motivational factors

## Abstract

Excessive time spent in sedentary behavior (SB) is associated with numerous health risks. These associations remain even after controlling for moderate-to-vigorous physical activity (PA) and body mass index, indicating that efforts to promote leisure time physical activity alone are insufficient. Cognitive and motivation variables represent potentially modifiable factors and have the potential of furthering our understanding of sedentary behavior. Hence, a systematic review was conducted to synthesize and critique the literature on the relationship between cognitive and motivational factors and sedentary behaviors. In April 2016, four electronic databases (Psych info, Pub Med, SPORTDiscus, Web of Science) were searched and a total of 4866 titles and abstracts were reviewed. After meeting inclusion criteria, study characteristics were extracted and the methodological quality of each study was assessed according to the Downs and Black Checklist. PRISMA guidelines for reporting of systematic reviews were followed. Twenty-five studies (16 cross-sectional, 8 longitudinal and one examining two populations and employing both a cross-sectional and prospective design) assessed 23 different cognitive and motivational factors. Seventeen studies were theory-based and 8 did not employ a theoretical model. Results showed that among SB-related cognitions, risk factors for greater sedentary time included having a more positive attitude towards SB, perceiving greater social support/norms for SB, reporting greater SB habits, having greater intentions to be sedentary, and having higher intrinsic, introjected, and external motivation towards SB. Protective factors associated with lower sedentary time included having greater feelings of self-efficacy/control over SB and greater intentions to reduce SB. Among PA-related cognitions, protective factors for lower SB included a more positive attitude towards PA, having greater social support/norms for PA, greater self-efficacy/control for PA, higher PA intentions, and higher intrinsic and identified motivation towards PA. In addition, feeling more supported and empowered in general was related with lower levels of SB. The average methodological quality score for included studies was 69% (SD = 9.15%; range 35–80%). In conclusion, a number of cognitive and motivational factors were identified that were associated with sedentarism. These findings have come from reasonably high quality studies. To further extend our understanding of the relation between cognitive and motivational factors and SB, more longitudinal, theory-driven studies examining cognitions and motivation from a sedentary perspective are required.

## 1. Introduction

Excessive time spent in sedentary behavior is associated with numerous health risks. An overview of 27 systematic reviews found that among adults, sedentary time is positively associated with all-cause mortality, fatal and non-fatal cardiovascular disease, type 2 diabetes, metabolic syndrome, and several types of cancers [1]. Among children and youth, the risks include obesity, increased blood pressure and total cholesterol, poorer self-esteem, social behavior problems, poorer physical fitness and lower academic achievement [1]. These associations remain even after controlling for moderate to vigorous physical activity and body mass index (BMI), indicating that efforts to promote leisure time physical activity alone are insufficient.

Sedentary behavior has been defined as “any waking behavior characterized by an energy expenditure ≤1.5 METs while in a sitting or reclining posture” [2]. Sedentary behaviors permeate all domains of life, including work, school, transportation, leisure/recreation, and spiritual/contemplative pursuits. The pervasiveness of sedentarism is evident through population-based studies, which indicate that Canadian and US adults spend an average of 9.7 and 7.7 hours per day, respectively, being sedentary [3],[4]. The high prevalence of sedentarism and its adverse outcomes has added a whole new paradigm to the physical activity field focused on understanding and reducing sedentary time.

Over the past few decades, there has been an increase in interest in ecological models as the guiding framework for understanding public health issues, including sedentary behavior [5],[6]. According to this approach, human health is viewed as the result of an interplay between a broad range of individual, social, environmental and policy factors [6]. At the individual level, intrapersonal factors such as psychological, biological, and demographical factors have been emphasized; social factors include those related to relationship, culture, and community; environmental factors refer to the organization, safety, attractiveness, and comfort of the physical environment; and policy factors refer to regulations, health care policies or incentives, the economic climate, and any governmental policies which have health implications [6]. Although ecological models emphasize the importance of intervening at multiple levels, a comprehensive understanding of the role of individual factors represents the first step towards a more complete appreciation of the issue in question. One such area of focus is the relationship between psychological factors and sedentary behavior.

Historically, psychological factors have been divided into three distinct faculties: affect, cognition, and conation [7]. The term “affect” refers to the emotional, or feeling aspects of human nature, and “cognition” refers to the rational, or intellectual aspects. “Conation” the third proposed part of the mind, is concerned with action, or volition, the mental effort and motivation required to carry out a proposed behavior [8]. Various formulations of the latter two aspects of psychological functioning are contained within current social-cognitive and motivational models of health behavior including the Health Belief Model [9], Theory of Reasoned Action [10], Theory of Planned Behavior (TPB) [11], Protection Motivation Theory (PMT) [12], Social Cognitive Theory [13], Health Action Process Approach (HAPA) [14], and Self Determination Theory (SDT) [15]. Individual constructs within these theories include attitudes, beliefs, knowledge, perceived barriers, self-efficacy, intention, and motivation. The link between these psychological variables and a number of health behaviors, including physical activity [16] is well established. Given the increased interest in sedentary behavior research, the aim of this systematic review was to synthesize and critique the current evidence on the association between cognitive and motivation factors and sedentary behavior and discuss avenues for future research.

The relationship between sedentary behavior and cognitive and motivational factors merits investigation for a number of reasons. First, even a cursory examination of a few studies examining cognitive factors and sedentary behavior shows that a significant link between the two does exist. For example, in a review on the correlates of sedentary behavior, Rhodes, Mark, and Temmel [17] identified several studies which found a significant relationship between psychological factors and sedentary time. At the same time, these authors pointed out the need for more research in this area and since their review was published in 2012, the number of studies examining cognitive factors has certainly grown. Second, cognitive and motivational constructs have proven to be useful for understanding numerous health-related behaviours such as physical activity [58]. Thus, it is likely that an examination of these factors also has the potential to increase our understanding of sedentary behavior. Third, while a number of published reviews have examined sedentary behavior correlates [5],[17]–[20], none have focused exclusively on psychological determinants from a cognitive and motivational perspective. As such, this review has the potential to identify gaps in the current research and significantly impact future research in this field. Fourth, in contrast to biological (e.g., genetic) or demographic determinants such as age, ethnicity, or socioeconomic status, cognition and motivation variables represent potentially modifiable protective or risk factors. Fifth and finally, while interventions aimed at reducing sedentary behavior are urgently needed, research to identify effective behavior change strategies cannot advance without a more complete understanding of the cognitive and motivational factors underpinning behavior change.

## 2. Method

This review was conducted according to PRISMA guidelines for transparent reporting of systematic reviews and meta-analyses [59]. A review of the literature was first carried out by searching the following separate, specific electronic databases from their inception (dates included wherever available in the databases) until May 10, 2016: PsycINFO, PubMed, SPORTDiscus, Web of Science. The keywords used referred to the exposure (cognitive, social-cognitive and motivation) and outcome (sedentary behavior) variables of interest. Specifically, the search strategy was agreed upon by SR, AG and HP and involved entering the following search terms into abovementioned pertinent databases: (sedentary OR sitting) AND (correlate OR predictor OR psychosocial OR theory OR social cognitive OR intention OR motivation OR attitude OR self-efficacy OR barriers OR beliefs). Ethical approval was not required since this was a review and did not involve human subjects. Next to the search in electronic databases, the authors' personal databases, previous published reviews, and references of included publications were checked. As this was the first systematic review to focus exclusively on the relationship between cognitive and motivational factors and sedentary behavior, the search was not limited to specific populations. For the purpose of this specifıc review, studies that involved populations of any age (e.g., children/youth, adolescents, adults, older adults) were included. After identification of studies through database searching, duplicate publications were removed. The titles and abstracts of all citations derived from the search were screened independently by two of the authors. In case of uncertainty to either include or exclude the study, the full paper was read. For all relevant publications, full-text articles were then read and assessed further for eligibility.

In order to be included in this review, studies had to meet the following criteria: (a) include one or more assessments of sedentary behavior or sedentary time; (b) examine the relationship of at least one cognitive or motivation variable with sedentary behavior or sedentary time; (c) be one of the following types of study: randomized controlled trials, cross-sectional studies, case-control studies and cohort studies (i.e., reviews, editorials and opinion articles were excluded since they did not contain primary data); and (d) be published in English. Studies were excluded if they measured sedentary time but failed to include possible correlates or if they did not measure predictors and behavior within the same individual (e.g., studies examining the relationship between parental beliefs and children's sedentary behavior were excluded). Studies examining mental health outcomes such as affect (e.g., depression, anxiety), quality of life, and physical self-perceptions were also excluded because these constructs are often viewed as consequences rather than antecedents of sedentary behavior. Finally studies that examined personality were excluded as they represent constructs that are considered stable and hence less modifiable.

All selected studies [21]–[45] were summarized in table format and data were extracted with regards to the author(s) and publication year, study population, sample size, sampling methods, study design, correlates/predictors examined, type and measurement of sedentary behavior or sedentary time, and the results pertaining to the relationship between behavior and significant correlates/predictors. In addition to summarizing the findings in table format and in text, we have visually represented the findings using what we have termed a pinwheel. The purpose of the pinwheel is to illustrate, at a glance, which constructs have been examined in the literature as well as whether a relationship emerged between the constructs. Within the health domain, sedentary behavior is considered a risk behavior. For this reason, the colour green was chosen to indicate a protective effect (i.e., lower sedentariness) due to its association with safety and the word “go-ahead” (e.g., its use in traffic lights). On the other hand, red is associated with a hazard and the word “stop”. For this reason, we used the colour red to indicate an association between a factor and increased sedentary behavior. Yellow was chosen to indicate a null effect due to the fact that it is seen as in-between green and red (e.g., on a traffic light signal).

The methodological quality of individual studies was assessed using the Downs and Black checklist [60]. The Downs and Black instrument assessed study quality including strength of reporting, external validity, internal validity (bias), internal validity (confounding), and power. The checklist consists of 27 items with a maximum score of 32 points. A modified version of the checklist was employed with items that were not relevant to non-experimental studies removed (8, 13–15, 17, 19, and 21–24). The adapted checklist consisted of 20 items, including 14 items from the original list (1–3, 6–7, 9–12, 16, 18, 20, and 25–26); three items that were modified (4, 5, and 27); and three items created for purposes of this review. Reporting items 4 and 5 from the original list were reworded to align with non-intervention (i.e., cross-sectional and prospective) studies being examined in this review. Item 27, concerning power from the original list was modified to address the number of participants needed to detect a significant association between an exposure and sedentary behavior. Of the three items created, two were internal validity criteria and one was concerned with study power. We believe that changes made to the original checklist had merit and that modifications held value in assessing the methodological quality of studies included in this review. Each quality criterion was rated as positive (1), negative (0), or unknown/insufficiently described (0). A positive sign (+) was given if the publication provided a sufficient description of the item, per the predefined criteria, and met the quality criteria for the item. A negative sign (−) was allotted if the publication did not provide an adequate description or did not address and/or perform the quality criteria for the item. Finally, if an insufficient or unclear description of the item was provided, a question mark (?) was given. The maximum possible score for the modified checklist was 20 points (higher scores indicate higher quality). The methodological quality of individual studies was independently scored by SR and verified by HP; if disagreements between assessors occurred, consensus was achieved through discussion with a third reviewer (AG). For each study, an overall methodological quality score was calculated. In addition, the percentage of studies meeting each quality criterion was calculated.

Data were not pooled for a number of reasons. First, there was little consistency among studies with respect to exposures and even when the same exposures were examined by multiple studies, they often used different scales. Second, studies used varying methodologies and reported statistics inconsistently. Therefore, to synthesize the evidence and allow conclusions to be drawn regarding the relationship between cognitive and motivational factors and sedentary behavior, a best-evidence synthesis that has been used in previous reviews [61] was implemented. The findings for each cognitive and motivational variable were interpreted on the following basis: there was no evidence of an association if more than 50% of the cross-sectional and prospective studies reported no association; there was inconclusive evidence for an association if 50% of the studies reported no association and 50% reported a positive or negative association; there was some evidence of an association if more than 50% of the studies reported a positive or negative association; and there was consistent evidence of an association if all of the studies reported a positive or negative association.

## 3. Results

**Figure 1.**
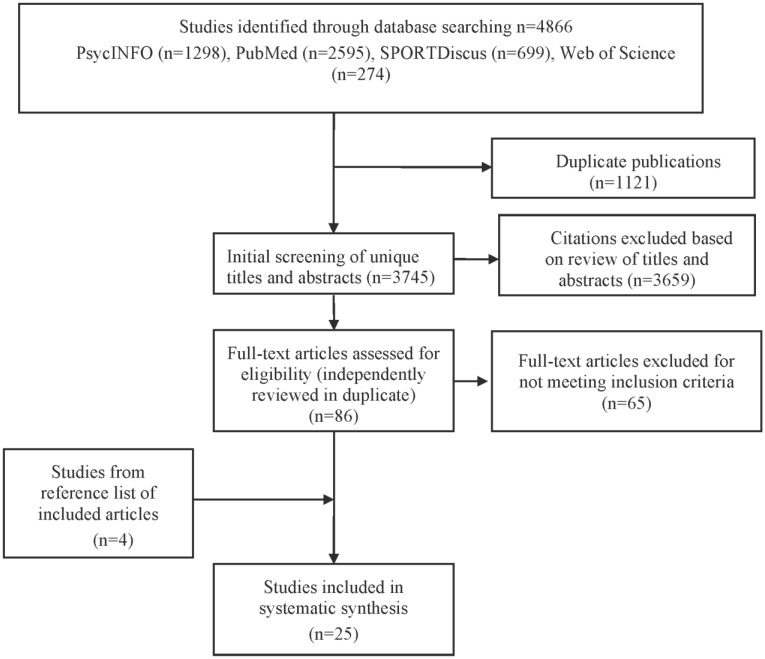
PRISMA flow diagram of study selection process in review of cognitive and motivational factors and sedentary behavior.

The electronic search produced 4,866 articles (1298 from PsycINFO, 2595 from PubMed, 699 from SPORTDiscus, and 274 from Web of Science; Figure 1). After removing duplicates (n = 1121), a total of 3745 publications remained. After titles and abstracts were examined, 86 full-text articles were read and assessed further for eligibility. Of those, 21 articles were identified as suitable. The reference lists of studies included for full-text review were then checked for additional relevant references, resulting in four additional studies. A total of 25 studies published between the years 2003 and 2016 met the inclusion criteria and were included in the review [21]–[45]. The characteristics of these studies are presented in Supplementary (Table S1).

Eight [21],[23],[26],[28],[29],[32],[34],[44] of the 25 reviewed studies did not specify a theoretical orientation in their study design and/or in the cognitive and motivational factors examined. Of these, only two [23],[28] were longitudinal or prospective in nature while the remaining six [21],[26],[29],[32],[34],[44] employed an observational, cross-sectional design. Researchers have emphasized the need for more longitudinal, prospective studies to be completed to fully understand temporal changes in sedentary time and corresponding psychological predictors [5],[17]. Five studies [21],[28],[29],[32],[34] examined sedentary behavior in children and/or adolescent populations whereas only three studies [23],[26],[44] investigated cognitive and motivational determinants of sedentary behavior in adult populations. Four studies [21],[28],[29],[34] employed convenience sampling methods and four studies [23],[26],[32],[44] used random sampling methods. Sample sizes ranged from 188 to 1,515 participants (M = 671.88, SD = 419.61). In terms of variables examined, six [23],[26],[28],[29],[32],[44] of the eight studies investigated correlates across multiple levels of influence (i.e., socio-demographic, physical environmental, social environmental, social-cognitive, psychosocial, health-related, work-related, behavioral) and two [21],[34] examined only cognitive variables. Furthermore, only four [23],[26],[34],[44] of the eight studies assessed cognitive factors from a sedentary perspective or in a sedentary-specific manner. One study [21] examined cognitive factors from a general point of view, while three studies [28],[29],[32] assessed the associations between physical activity and/or exercise-specific cognitive factors and sedentary behavior.

Regarding measurement of sedentary behavior, all eight studies employed self-report measurement tools with only one study [21] capturing sedentary behavior both through self-report and objective measures. Despite the majority of studies measuring self-reported sedentary behavior, there was inconsistency between them in terms of specific sedentary pursuits assessed and the domains observed. One study [21] examined total time spent sedentary and time spent in specific leisure sedentary activities; one study [23] investigated determinants of context-specific sedentary time; four studies [28],[29],[32],[34] measured screen time and/or screen-based behaviors; and two studies [26],[44] looked at either occupational or work-related sitting time.

**Figure 2.**
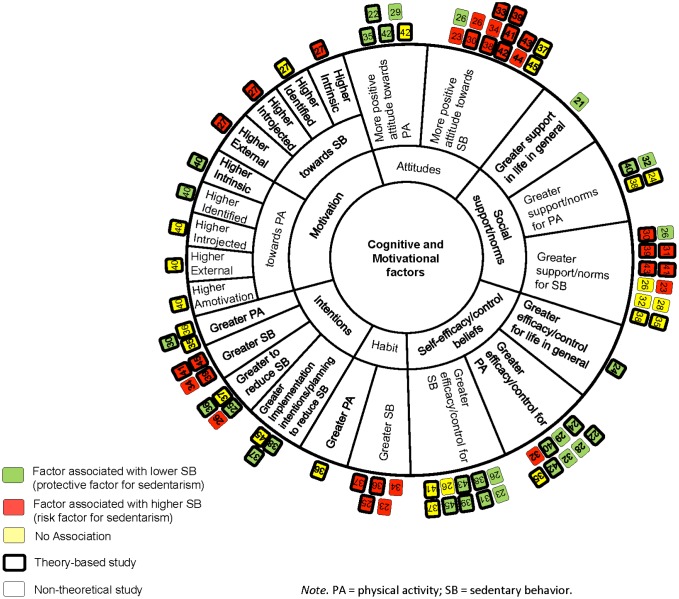
Pinwheel showing the association of cognitive and motivational factors with sedentary behavior.

Primary associations of cognitive and motivational factors with sedentary behavior examined through non-theoretical studies are summarized in Table S1 and illustrated in Figure 2. Overall, the associations reported in Table S1 were small to medium in size. Five studies [23],[26],[29],[34],[44] investigated the relationship between attitudes and sedentary behavior. Of these, one study [29] found more positive attitudes towards exercise to be associated with lower sedentary behavior. Four studies [23],[26],[34],[44] found more positive attitudes towards sedentary behavior to be associated with higher sedentary behavior. Contrary to expectations, one study [26] found more positive attitudes towards sedentary behavior to be associated with lower sedentary behavior. Five studies [21],[23],[26],[28],[32] examined the relationship between social support and/or norms and sedentary behavior. One study [21] found greater support in life to be associated with lower sedentary behavior, while one [32] study found greater support for physical activity to be associated with lower sedentary behavior. Three studies [26],[28],[32] found no association between sedentary behavior and greater support and/or norms for sedentary behavior. However, one study [26] found greater norms for sedentary behavior to be associated with lower sedentary behavior and one study [23] found greater support and/or norms to be associated with higher sedentary behavior. Five studies [23],[26],[28],[29],[32] investigated the relationship between self-efficacy and/or control beliefs and sedentary behavior. Two studies [28],[29] found greater self-efficacy for physical activity to be associated with lower sedentary behavior, while one study [32] found this factor to be associated with lower sedentary behavior for boys but higher sedentary behavior for girls. One study [23] found greater self-efficacy for sedentary behavior to be associated with lower sedentary behavior and one study [26] found greater control for sedentary behavior to be associated with lower sedentary behavior. One study [26] showed no association between sedentary behavior and self-efficacy for sedentary behavior. Two studies [23],[34] examined the relationship between sedentary behavior habits and sedentary behavior, both of which found greater sedentary behavior habits to be associated with higher sedentary behavior. Two studies [26],[34] investigated the relationship between intentions and sedentary behavior. One study [34] reported greater sedentary behavior intentions to be associated with higher sedentary behavior. Contrary to expectations, one study [26] found greater intentions to reduce sedentary behavior to be associated with higher sedentary behavior.

Of the 25 studies included in this review, 17 were theoretically driven in their approach (see Table S1). Of these, 10 studies [22],[24],[27],[30],[31],[38],[39],[40],[42],[43] employed an observational, cross-sectional design and six [25],[33],[35],[36],[37],[45] were longitudinal, prospective in nature. One study [41] included samples from two separate populations, and employed both cross-sectional and prospective designs. Timelines for prospective studies ranged from seven days to three years. Five studies [22],[30],[31],[33],[38] examined sedentary behavior in children and/or adolescent populations, five studies [25],[27],[36],[40],[45] examined factors associated with sedentary behavior in college and/or university student populations, and six studies [24],[35],[37],[39],[42],[43] investigated determinants of sedentary behavior in adult populations. One study [41] investigated sedentary behavior in two samples including an adult population and a university student population. Twelve studies [22],[24],[25],[27],[31],[35]–[40],[45] employed convenience sampling methods, four studies [30],[33],[42],[43] used random sampling methods, and one study [41] employed both. Sample sizes ranged from 31 to 1,552 participants (M = 520, SD = 410.35). With regards to determinants examined, four studies [24],[33],[38],[43] investigated factors across multiple levels of influence (i.e., socio-demographic, physical environmental, social environmental, social-cognitive, psychosocial, health-related, work-related, behavioral), seven studies [22],[25],[30],[31],[36],[37],[42] examined cognitive variables only, and six [27],[35],[39],[40],[41],[45] were grounded in prominent social-cognitive and motivational theoretical models, such as Theory of Planned Behavior (TPB) [11], Protection Motivation Theory (PMT) [12], and Self-Determination Theory (SDT) [15]. Furthermore, 11 of the 17 studies [25],[27],[30],[31],[33],[37]–[39],[41],[43],[45] assessed cognitive and motivational factors from a sedentary perspective or in a sedentary-specific manner whereas four studies [22],[24],[35],[40] assessed physical activity related factors and two studies [36],[42] examined factors from both a sedentary and physical activity perspective.

In terms of sedentary behavior measurement, the majority of studies employed self-report measurement tools, however, two studies [33],[35] measured sedentary behavior objectively and two studies [25],[37] captured sedentary behavior both through self-report and objective measures. Nine studies [22],[24],[25],[33],[35],[36],[37],[38],[40] measured total sedentary time or overall sedentary behavior; five studies [27],[39],[41],[42],[45] investigated determinants of context-specific sedentary time; and three studies [30],[31],[43] measured screen time and/or screen-based behaviors.

Cognitive and motivational factors grounded in a theory-based framework and their respective associations to sedentary behavior are summarized in Table S1 and illustrated in Figure 2. Overall, the associations reported in Table S1 were small to medium in size. Eleven studies [22],[30],[33],[35],[37]–[39],[41]–[43],[45] examined the relationship between attitudes and sedentary behavior. Three studies [22],[35],[42] found more positive attitudes towards physical activity to be associated with lower sedentary behavior, however, one study [42] found no association between this factor and sedentary behavior. Seven studies [30],[33],[38],[39],[41]–[43] found more positive attitudes towards sedentary behavior to be associated with higher sedentary behavior, however, two studies [37],[45] found no association.

Nine studies [24],[30],[31],[35],[38]–[41],[43] investigated the relationship between social support and/or norms and sedentary behavior. One study [40] found greater support for physical activity to be associated with lower sedentary behavior; however, two studies [24], [38] failed to show an association. Five studies [30],[31],[39],[41],[43] found greater support and/or norms for sedentary behavior to be associated with higher sedentary behavior. Two studies [35],[38] reported no association between this factor and behavior.

Twelve studies [22],[24],[31],[35],[37]–[43],[45] examined the relationship between self-efficacy and/or control beliefs and sedentary behavior. One study [24] found that greater efficacy and control for life in general was associated with lower sedentary behavior. Four studies [22],[24],[40],[42] found greater self-efficacy and/or control beliefs for physical activity to be associated with lower sedentary behavior, while one study [35] found no association. Five studies [31],[38],[39],[43],[45] reported that greater self-efficacy and/or control for sedentary behavior was associated with lower sedentary behavior; however, two studies [37],[41] failed to show an association between this factor and sedentary behavior.

Three studies [25],[36],[37] investigated the relationship between habits, either towards sedentary behavior or physical activity, and sedentary behavior. Three studies [25],[36],[37] found greater sedentary behavior habits to be associated with higher sedentary behavior. One study [36] failed to show an association between greater physical activity habits and sedentary behavior.

Nine studies [25],[30],[35]–[39],[41],[45] examined the relationship between intentions and sedentary behavior. Two studies [37],[38] found greater implementations intentions and/or planning to reduce sedentary behavior to be associated with lower sedentary behavior; however, one study [45] found no association. Two studies [25],[36] found greater intentions to reduce sedentary behavior to be associated with lower sedentary behavior. One study [37] showed no association between this factor and behavior. Three studies [39],[41],[45] found greater sedentary behavior intentions to be associated with higher sedentary behavior. One study [30] found greater physical activity intentions to be associated with lower sedentary behavior; however, two studies [35],[36] failed to show an association.

Two studies [27],[40] investigated the relationship between motivational factors and sedentary behavior. One study [40] found higher intrinsic motivation and identified regulation towards physical activity to be associated with lower sedentary behavior. However, no associations were found between introjected regulation, external regulation, or amotivation and sedentary behavior. One study [27] found higher intrinsic motivation, introjected regulation, and external regulation towards sedentary behavior to be associated with higher sedentary behavior. In this study, no association was found between identified regulation towards sedentarism and behavior.

The modified Downs and Black checklist for assessment of the methodological quality of reviewed studies, including the percentage of studies meeting each item, is presented in Table 1. The overall scores of the quality assessment for each study are presented in Table 2. When the studies were evaluated, the methodological quality score of the publications ranged from 35% to 80%. The average quality score for included studies was 69% (SD = 9.15). Out of the 25 publications (26 reported studies), one study [34] had a score of less than 50%. Three studies [22],[31],[36] had a score of 60%, eight studies [21],[24],[27],[29],[30],[35],[39],[40] had a score of 65%, three studies [38,41b,45] had a score of 70%, eight studies [26,28,32,33,41a,42–44] had a score of 75%, and three studies [23],[25],[37] had a score of 80%. The average score of the included studies for the quality sub-scales of reporting, external validity, internal validity, and power were 88%, 31%, 71%, and 12%, respectively. Also highlighted through the assessment was the percentage of studies meeting each item on the checklist (Table 1). The majority of studies satisfied the reporting criteria (items 1–9) with >80% of studies meeting each of the items 1–8. However, only 42% of studies reported actual probability values for the main outcomes except where the probability value is less than 0.001 (item 9). In terms of the external validity criteria, items 10 and 11 attempt to address the representativeness of the findings of the study and whether they may be generalized to the population from which the study subjects were derived. Only 35% and 27% of studies met these items, respectively. The proportion of studies meeting the quality items with respect to internal validity (items 12–18) varied considerably per item, with only 35% of studies measuring the cognitive and/or motivation variables at a time prior to the assessment of sedentary behavior (item 13). Further, only 12% of studies scored positive on item 16 and included an objective assessment or some corroboration of the objective and subjective assessment in the measurement of sedentary behavior. For the power criteria (items 19–20), 88% of studies did not report a formal power calculation for determining the association between an exposure and sedentary behaviors (item 19). Because of this, it was unknown whether the sample size used for analysis was sufficiently powered for these studies (item 20).

**Table 1.** Checklist for Assessment of the Methodological Quality of Cross-sectional and Prospective Studies [based on modified Downs and Black checklist].

Criteria (rating of criteria: + = yes, – = no, ? = not or insufficiently described)	% studies meeting the item
***Reporting***	
1. Is the hypothesis/aim/objective of the study clearly described?	100
2. Are the main outcomes to be measured clearly described in the Introduction or Methods section?	100
3. Are the characteristics of the participants included in the study clearly described?	100
4. Is the study design clearly described (i.e., cross-sectional vs. prospective; if prospective, time of assessments)?	89
5. When appropriate, were principal covariates clearly described?	81
6. Are the main findings of the study clearly described?	100
7. Does the study provide estimates of the random variability in the data for the main outcomes?	92
8. Have the characteristics of participants lost to follow-up and/or with missing data been described?	89
9. Have actual probability values been reported (e.g. 0.035 rather than <0.05) for the main outcomes except where the probability value is less than 0.001?	42
***External Validity***	
10. Were the subjects asked to participate in the study representative of the entire population from which they were recruited?	35
11. Were those subjects who were prepared to participate representative of the entire population from which they were recruited?	27
***Internal Validity—bias***	
12. If any of the results of the study were based on “data dredging”, was this made clear?	100
13. Were the exposure variables assessed at a time prior to the measurement of sedentary behavior?	35
14. Were the statistical tests used to assess the main outcomes appropriate?	100
15. Were the main outcome measures used accurate (valid and reliable)?	96
16. Did measurement of sedentary behavior (outcome) include an objective assessment or some corroboration of the objective and subjective assessment?	12
***Internal validity—confounding (selection bias)***	
17. When appropriate, was there adequate adjustment for confounding (i.e., covariates) in the analyses from which the main findings were drawn?	81
18. Were losses of participants to follow-up and/or with missing data taken into account?	73
***Power***	
19. Did the study report a formal power calculation for determining the association between an exposure and sedentary behaviors?	12
20. Was the sample size used for analyses reflective of the power calculation?	12

**Table 2.** Overall scores of the methodological quality assessment for the included studies.

Author/Criteria (1–20)	1	2	3	4	5	6	7	8	9	10	11	12	13	14	15	16	17	18	19	20	Total/%
[21] Atkin, Corder, Goodyer, et al., 2015	+	+	+	+	+	+	+	+	−	−	?	+	−	+	+	−	+	+	−	?	13 65%
[22] Bai, Chen, Vazou, et al., 2015	+	+	+	−	+	+	+	+	−	−	−	+	−	+	+	−	+	+	−	?	12 60%
[23] Busschaert, De Bourdeaudhuij, Van Cauwenberg, et al., 2016	+	+	+	+	+	+	+	+	−	+	+	+	+	+	+	−	+	+	−	?	16 80%
[24] Chang & Sok, 2015	+	+	+	+	−	+	+	+	+	−	?	+	−	+	+	−	−	−	+	+	13 65%
[25] Conroy, Maher, Elavsky, et al., 2013	+	+	+	+	+	+	+	+	+	−	−	+	+	+	+	+	+	+	−	?	16 80%
[26] De Cocker, Duncan, Short, et al., 2014	+	+	+	−	+	+	+	+	+	+	+	+	−	+	+	−	+	+	−	?	15 75%
[27] Gaston, De Jesus, Markland, et al., 2016	+	+	+	+	+	+	+	+	−	−	?	+	−	+	+	−	+	+	−	?	13 65%
[28] Gebremariam, Totland, Andersen, et al., 2012	+	+	+	+	+	+	+	+	+	−	?	+	+	+	+	−	+	+	−	?	15 75%
[29] Ham, Sung, & Kim, 2013	+	+	+	+	−	+	+	+	+	−	?	+	−	+	+	−	−	−	+	+	13 65%
[30] He, Piché, Beynon, et al., 2010	+	+	+	+	+	+	−	+	−	+	+	+	−	+	+	−	+	−	−	?	13 65%
[31] Hoyos Cillero, Jago, & Sebire, 2011	+	+	+	+	+	+	+	−	+	−	?	+	−	+	+	−	+	−	−	?	12 60%
[32] Huang, Wong, & Salmon, 2013	+	+	+	+	+	+	+	+	−	+	+	+	−	+	+	−	+	+	−	?	15 75%
[33] Janssen, Basterfield, Parkinson, et al., 2015	+	+	+	+	−	+	+	+	−	+	?	+	+	+	+	−	−	+	+	+	15 75%
[34] Kremers & Brug, 2008	+	+	+	+	−	+	−	−	−	−	−	+	−	+	−	−	−	−	−	?	735%
[35] Lowe, Danielson, Beaumont, et al., 2015	+	+	+	−	+	+	+	−	+	−	−	+	+	+	+	+	+	−	−	?	13 65%
[36] Maher & Conroy, 2015	+	+	+	+	−	+	+	+	−	−	−	+	+	+	+	−	−	+	−	?	12 60%
[37] Maher & Conroy, 2016	+	+	+	+	+	+	+	+	+	−	?	+	+	+	+	+	+	+	−	?	16 80%
[38] Norman, Schmid, Sallis, et al., 2005	+	+	+	+	+	+	+	+	+	−	?	+	−	+	+	−	+	+	−	?	14 70%
[39] Prapavessis, Gaston, & DeJesus, 2015	+	+	+	+	+	+	+	+	−	−	−	+	−	+	+	−	+	+	−	?	13 65%
[40] Quartiroli & Maeda, 2014	+	+	+	+	+	+	+	+	−	−	?	+	−	+	+	−	+	+	−	?	13 65%
[41] Rhodes & Dean, 2009 (A)	+	+	+	+	+	+	+	+	−	+	+	+	−	+	+	−	+	+	−	?	15 75%
[41] Rhodes & Dean, 2009 (B)	+	+	+	+	+	+	+	+	−	−	−	+	+	+	+	−	+	+	−	?	14 70%
[42] Salmon, Owen, Crawford, et al., 2003	+	+	+	+	+	+	+	+	−	+	+	+	−	+	+	−	+	+	−	?	15 75%
[43] Van Dyck, Cardon, Deforche, et al., 2011	+	+	+	+	+	+	+	+	+	+	−	+	−	+	+	−	+	+	−	?	15 75%
[44] Wallmann-Sperlich, Bucksch, Schneider, et al., 2014	+	+	+	+	+	+	+	+	+	+	+	+	−	+	+	−	+	−	−	?	15 75%
[45] Wong, Gaston, DeJesus, et al., 2016	+	+	+	+	+	+	+	+	−	−	−	+	+	+	+	−	+	+	−	?	14 70%

## 4. Discussion

The purpose of this paper was to systematically review and critique the current literature on the role that cognitive and motivational processes play in understanding sedentary behavior. While other reviews have been conducted on socio-demographic and behavioral correlates of sedentary behavior, to our knowledge this is the first to focus exclusively on cognitive and motivational factors.

Primary associations of cognitive and motivational factors with sedentary behavior examined through non-theoretical studies [21],[23],[26],[28],[29],[32],[34],[44] showed that among children and adolescents, a more positive attitude towards watching TV and using a computer [34], a less positive attitude towards exercise [29], greater habit strength for watching TV and using a computer [34], and greater intentions for sedentary behavior [34] were associated with greater time spent in sedentary pursuits. Conversely, a more negative attitude towards screen time [34], a more positive attitude towards exercise [29], greater perceived family and peer support for physical activity [32], better friendship quality [21], greater perceived family functioning [21], and greater self-efficacy to engage in physical activity and overcome barriers [28],[29],[32] were associated with lower sedentary behavior. It is worth nothing that the majority of studies (4 out of 5) [28],[29],[32],[34] with children and adolescents specifically examined screen-related sedentary behaviours. This is consistent with findings from past reviews, which found a less-developed research base on correlates of sedentary behavior among adults and highlighted the need to address this issue [5],[17].

Among adults, one study [44] found, for men only, that a more positive attitude towards sitting, measured as indifference towards sitting for long periods of time, was associated with increasing work-related sitting durations. De Cocker and colleagues [26] sought to identity socio-demographic, health-related, work-related and psychosocial correlates of occupational sitting in Australian adult employees. It was found that adults who perceived greater control over how much they sat reported lower occupational sitting time, whereas those who believed that reducing their sitting time would be disadvantageous reported higher occupational sitting time. No associations emerged between self-efficacy or social support to sit less in the next month at work and occupational sitting time. Contrary to expectations, De Cocker and colleagues found that adults who perceived higher social norms towards sitting less at work, reported greater benefits of sitting less, and had greater intentions to sit less at work reported higher occupational sitting time compared to respective comparison counterparts. They also found that employment status and occupational classification had a moderating effect on the association between control to sit less at work and occupational sitting time such that lack of control to sit less at work was positively associated with occupational sitting time among full- and part-time workers and white-collar and professional workers only. These findings suggest that those who are full-time, white-collar and/or professional workers may have positive attitudes towards sitting less and intentions to sit less; however, these individuals are also more likely to be employed in jobs that require prolonged sitting. Thus, in the absence of control, even attitudes and intentions are insufficient to lead to reduced sedentary behavior.

In a longitudinal study, Busschaert and colleagues [23] examined the relationship between changes in social-cognitive variables from baseline to one-year follow-up with changes in context-specific sitting times. They found that positive attitudes towards watching TV and computer use was associated with more sitting while watching TV and more sitting while using a computer, respectively. Higher perceived modeling of sedentary behavior (i.e., time partner spends watching TV) was associated with more sitting while watching TV and higher norms associated with computer use and motorized transport was associated with more sitting in those contexts. Self-efficacy to reduce computer use was associated with less sitting time while using a computer, whereas self-efficacy to use active transportation was associated with less sitting during motorized transport. In contrast to De Cocker and colleagues [26], Busschaert et al.'s [23] findings are in line with the expected relationships between cognitive variables and behavior. The most likely reason for this difference is De Cocker et al. [26] examined occupational sitting, a type of sedentary behavior less under an individual's control, while Busschaert et al. [23] examined leisure time sitting.

For the cognitive factors examined through non-theoretical studies, there is: consistent evidence of an unfavorable association between positive attitudes towards sedentary behavior, sedentary habits, sedentary intentions, and time spent in sedentary pursuits; consistent evidence of a favorable association between positive attitudes towards physical activity, general social support, support/norms for physical activity, and sedentary behavior; some evidence of a favorable association between self-efficacy/control beliefs for sedentary behavior and time spent in sedentary pursuits; and no evidence of an association between support/norms for sedentary behavior and levels of sedentary behavior (see Table S1 and Figure 2). While there was consistent evidence of an association between self-efficacy/control for physical activity and levels of sedentary behavior with majority of studies indicating a favorable association, one study demonstrated an unfavorable association between this factor and behavior. It is important to note that sedentary intentions, attitudes towards physical activity, general social support, and support/norms for physical activity and their relationship with sedentary behavior were only examined in one non-theoretical study each.

Health behavior change scientists from numerous fields, including physical activity, have underscored the superiority of using theory to guide their research [46]. Studies investigating cognitive and motivational factors grounded in a theory-based framework and their respective associations to sedentary behavior are summarized in Table S1 and Figure 2. Attitude, either towards sedentary behavior or physical activity, was one of the most often studied cognitions with 11 studies [22],[30],[33],[35],[37]–[39],[41]–[43],[45] including at least one measure of this construct. Seven studies [30],[33],[38],[39],[41]–[43] revealed that having more positive attitudes towards sedentary behavior was associated with higher levels of sedentary behavior while two studies [37],[45] showed no association between these constructs. Three studies [22],[35],[42] demonstrated having more positive attitudes towards physical activity to be associated with lower levels of sedentary behavior; whereas, one study [42] showed no association between these constructs. These findings are largely consistent with the bulk of the research on the relation between attitude and behavior, which shows that attitude can be a strong predictor of behavior [47]. A common strength of the included studies was the assessment of attitudes towards a single, specific, well-defined behavior. This may be one reason why the majority of studies demonstrated significant findings. Attitude can refer to affective attitudes (e.g., enjoyment of sitting) or instrumental attitudes (e.g., pros or cons associated with sedentary behavior). Among the studies included, three [30],[33],[42] assessed only affective attitudes, three [37],[43],[45] assessed only instrumental attitudes, and two [38],[39] assessed both affective and instrumental attitudes. Among studies examining attitudes towards physical activity, two studies [22],[42] examined affective and one study [35] examined both. For sedentary attitudes, all affective attitude measures and three out of the five instrumental attitude measures significantly predicted behavior. For physical activity attitudes, three out of four measures of affective attitudes and the only instrumental attitude measure were significant correlates of behavior. Taken together, these findings indicate that how individuals *feel* about sedentary behavior, and, to a lower extent physical activity, plays a strong role in affecting how sedentary they are. In summary, there is some evidence of an unfavorable association between positive attitudes towards sedentary behaviors and time spent in sedentary pursuits. There also is some evidence of a favorable association between positive attitudes towards physical activity and levels of sedentary behavior.

With regards to social support and norms as potential factors related to sedentary behavior, five studies [30],[31],[39],[41],[43] demonstrated that greater support/norms for sedentary behavior were associated with higher sedentary behavior. Two studies [35],[38] failed to show an association between these factors and sedentary behavior. Five of these [31],[35],[39],[41],[43] specifically explored the influence of norms towards sedentary behavior as a potential risk factor. For the most part, the results highlight the importance of subjective norms in understanding levels of sedentary behavior. Prapavessis and colleagues [39] suggested that, as the majority of adults spend far more time being sedentary than being active, the role of others appears to be more important in encouraging sedentary than physical activity pursuits. Additionally, decisions to be sedentary are likely to be socially motivated, and socially motivated decisions enhance the recognition of normative perceptions, which in turn may influence behavior through intentions [48]. One study [40] found that greater support/norms for physical activity was associated with lower sedentary behavior; however, two studies [24],[38] found no association between this factor and behavior. Among the studies, which failed to show an association, Chang and Sok [24] examined the relationship between social support for physical activity and sedentary behavior in elderly persons with hypertension and Norman and colleagues [38] examined parent-directed support for physical activity and sedentary behavior in a sample of adolescents. Chang and Sok [24] suggested, from their findings, that predictors of sedentary behavior might be distinct from the well-known powerful predictors of physical activity. Quartiroli and Maeda [40], however, found that scoring higher with respect to the basic psychological need of relatedness in exercise was associated with lower levels of sedentary behavior. It is proposed then that perhaps, the perception of being close and connected to others through physical activity (i.e., relatedness) is a determinant of sedentarism to be explored further. In summary, there is some evidence of an unfavorable association between support/norms for sedentary behavior and time spent in sedentary pursuits. However, presently there is no clear evidence of an association between support/norms for physical activity and levels of sedentary behavior.

In terms of self-efficacy/control beliefs, outcomes assessed included self-efficacy to reduce sedentary behavior and/or screen time, scheduling self-efficacy, response self-efficacy, and perceived behavior control. Five studies [31],[38],[39],[43],[45] showed that greater self-efficacy/control for sedentary behavior was associated with lower sedentary behavior while two studies [37],[41] showed no association. Maher and colleagues [37] failed to show an association between self-efficacy to limit sedentary behavior and sedentary time in older adults; however, task self-efficacy was associated with intentions to limit sedentary behavior. This indicates that efficacy beliefs may be an indirect determinant of sitting time in older adults. The authors also suggested that older adults might have particularly low levels of task self-efficacy to limit sedentary behavior due to pain or functional limitations, aging stereotypes, and previous failed attempts to engage in physical activity. Rhodes and Dean [41] showed no association between perceived behavioral control and sedentary leisure behaviors; this is contrary to findings by Prapavessis and colleagues [39] who found perceived behavioral control to be a protective factor for sedentarism. Rhodes and Dean [41] acknowledged that the absence of perceived behavioral control as a behavioral correlate or even an independent predictor of intention is markedly different from most health behaviors. However, they indicated that this could offer important information on the discriminant motivational structure of sedentary leisure behaviors compared to what is known about a behavior like physical activity, and suggest the difference may be due to high access and ease of use among people who wish to perform these behaviors. Additionally, four studies [22],[24],[40],[42] showed that greater self-efficacy and control for physical activity was associated with lower sedentary behavior; however, one study [35] found no association between sedentary time and greater efficacy/control beliefs towards physical activity. This study was markedly different from the other studies in that it was examining TPB correlates of sedentary behavior in cancer patients with brain metastases. In this population, attitudes towards physical activity were most strongly correlated with sedentary behavior. The authors indicated that although not statistically significant, there were potentially meaningful differences in perceived behavioral control between those who sit or supine less than 20.7 hours per day and those who accumulate 20.7 hours or greater. One study [24] found that feeling more empowered overall (i.e., having greater feelings of efficacy and control for life in general) was associated with lower levels of sedentarism. In summary, there is some evidence of a favorable association between self-efficacy/control for sedentary behavior and time spent in sedentary pursuits. Likewise, there is some evidence of a favorable association between self-efficacy/control for physical activity and levels of sedentary behavior. There is also consistent evidence of a favorable association between self-efficacy/control for life in general and levels of sedentary behavior; however, caution is warranted when interpreting this finding as only one study to date has examined this factor in relation to sedentary behavior.

Recently, due to the sporadic, varied, and unstructured nature of sedentary behavior, researchers have suggested that habit formation may play a role in understanding sedentary pursuits [36],[37]. Dual process theories of motivation propose that both controlled and automatic motivational processes regulate behavior. Controlled processes are conscious, reflective, and volitional and include many of the constructs outlined in social-cognitive theories and this review. Automatic processes, on the other hand, are non-conscious, reflexive, and unintended, and can include constructs such as habits. It has been suggested that these two motivational processes may operate independently or interact to regulate health behaviors [37]. Habits develop through the repeated pairing of a contextual cue with behavior, over time, until the contextual cue automatically elicits the behavioral response [49]. Three studies [25],[36],[37] included in this review found greater sedentary behavior habits to be a risk factor for sedentarism. Maher and Conroy [37] recently showed that habit strength for sedentary behavior was the greatest of all the predictors of behavior, demonstrating that automatic processes, such as habits, represent a crucial component in understanding sedentarism. The findings of these studies demonstrated that the association between habit strength and sedentary behavior appears to be robust for both young and older adults. On the other hand, one study [36] failed to show an association between greater physical activity habits and sedentary behavior. The role of both controlled and automatic motivational processes in regulating sedentary behavior needs to be examined further. Dual-process models incorporating habit formation (i.e., automatic and unreasoned process) into prominent social-cognitive theoretical frameworks could explain a greater proportion of sedentary behavior and be effective in sedentary behavior reduction efforts. There has also been a call for improved measures of habit processes within the health domain, and specifically that of sedentarism [50],[37]. Grove and Zillich [50] proposed a theoretical model of psychological processes associated with habitual exercise, in which they suggest that habitual health behaviours are characterized by several common features, including; strong stimulus response (S-R) bonds (i.e., driven by cues), automaticity, patterning of action, and negative consequences for nonperformance. It is possible that this model may hold value for assessing habits related to sedentary behavior. In summary, there is consistent evidence of an unfavorable association between sedentary behavior habits and time spent in sedentary pursuits, however, there is no evidence of an association between physical activity habits and levels of sedentary behavior.

In many behavior change models, intentions are seen as the principal, predisposing factor as to whether someone will engage in a particular health behavior (or not). With regards to intention as a potential factor associated with sedentary behavior, one study [30] found greater physical activity intentions to be a protective factor for sedentarism; however, two studies [35],[36] found no association. Two studies [25],[36] demonstrated having greater intentions to reduce sedentary behavior to be associated with lower sedentary behavior. In one study [37], no association was found. In terms of intentions as risk factors for sedentarism, three studies [39],[41],[45] found greater sedentary behavior intentions to be associated with higher sedentary behavior. Finally, two studies [37],[38] showed greater implementation intentions or planning to reduce sedentary behavior to be associated with less sedentary behavior, while one study [45] found no association. The abovementioned studies, taken together, provide evidence to support the theoretical construct of both goal and implementation intentions as correlates of sedentary behavior and suggest that engagement in sedentary pursuits may be a controlled motivational process similar to other health behaviours. Future studies examining the role of sedentary goal intentions need to be conducted to determine whether measuring goal intentions towards sedentary behavior itself, or goal intentions to change sedentary behavior is a more viable approach. In summary, there is no clear evidence of a favorable association between physical activity intentions and levels of sedentary behavior. However, there is consistent evidence of an unfavorable association between sedentary behavior intentions and time spent in sedentary pursuits. Additionally, there is some evidence of a favorable association between intentions to reduce sedentary behavior and levels of sedentary behavior. There is also some evidence of a favorable association between implementation intentions and/or planning to reduce sedentary behavior and levels of sedentary behavior.

Two studies [27],[40] examined motivation type within a Self Determination Theory framework as a potential psychological determinant of sedentary behavior. Gaston, De Jesus, Markland, and Prapavessis [27] demonstrated higher external regulation, higher introjected regulation, and high intrinsic motivation towards sedentary behavior to be risk factors for sedentarism. Specifically, Gaston and colleagues found that intrinsic motivation was the strongest predictor of sedentary behavior, followed by external regulation and introjected regulation. These authors examined leisure and work/school activities separately, and found that autonomous motives (i.e., intrinsic motivation) underlied leisure/recreation sedentary pursuits whereas more controlled motives (i.e., external and introjected regulation) influenced work/school sedentary activities. Identified regulation, which occurs when an individual recognizes that a behavior is beneficial for achieving a personally valued goal and consequently adopts the behavior as their own [27], was not related to behavior. Since sitting is typically engaged in not for its own sake but as a means to an end, this finding was surprising. It should also be recognized that this study was the first to adapt the Behavioral Regulation in Exercise Questionnaire (BREQ) [51] for sedentary behavior. Quartiroli and Maeda [40] showed higher intrinsic motivation and higher identified regulation towards physical activity to be associated with lower levels of sedentary behavior. No association was found for introjected regulation, external regulation, and amotivation towards physical activity and sedentary behavior. The finding in both studies that intrinsic motivation is related with sedentary behavior is consistent with the relation on attitudes and behavior. Similarly to measures of affective attitude, intrinsic motivation refers to performing a behavior for its own sake, in other words, for the enjoyment of it. More studies are required to validate the theoretical structure of SDT in explaining sedentary behavior and to identify sedentary-specific motivational factors related to sedentarism. In summary, there is convincing evidence from one study [40] of a favorable association between intrinsic motivation and identified regulation towards physical activity and levels of sedentary behavior. However, there is no evidence of an association between introjected regulation, external regulation, and amotivation towards physical activity and sedentary behavior. There is also convincing evidence from one study [27] of an unfavorable association between external regulation, introjected regulation, and intrinsic motivation towards sedentary behavior and time spent in sedentary pursuits. No evidence of an association between identified regulation towards sedentary behavior and levels of sedentary behavior has been shown.

Given that the associations between cognitive factors, motivational factors and sedentary behavior or sedentary time were small to medium in size, researchers interested in targeting these modifiable variables will need to take this into consideration when using these as agents of change for sedentary behavior interventions. Furthermore, these findings suggest that both physical activity related and sedentary-specific cognitive and motivational factors will play a role in understanding sedentarism. With respect to movement-related factors, research has shown a strong, inverse correlation between sedentary behavior and light-intensity physical activity [62], as well as a small to medium inverse correlation between sedentary behavior and leisure time physical activity [17],[63]. If these behaviors are associated with one another, then it is highly likely that physical activity related cognitions could be associated with time spent sedentary. The findings, herein, serve to confirm this rationale and demonstrate that physical activity related cognitive and motivational factors are correlates of sedentary behavior. In order to maximize the contribution of studies examining physical activity related factors to our understanding of sedentary behavior determinants; researchers might need to measure these cognitions as they pertain to specific types of physical activity (i.e., total physical activity, light-intensity physical activity).

Based on the Downs and Black checklist [60] for assessment of the methodological quality, the findings from the included studies in this systematic review come from reasonably high quality studies (see Tables 1 and 2). For instance, 22 of the 26 reported studies had overall quality scores ≥65% and 11 of the 26 studies had overall quality scores ≥75%. We found no difference between the average quality scores (i.e., percentages) of theoretically-driven (M = 68.9%, SD = 6.4) versus non-theory based studies (M = 68.1%, SD = 13.5). Furthermore, studies that demonstrated an association between cognitive and/or motivational variables and sedentary behavior (M = 69%, SD = 9.2) were of similar quality to those studies that found no association between these constructs (M = 71%, SD = 5.8). The two major weaknesses with the included studies are that: only 35% of them measured the cognitive and/or motivational variables prior to the assessment of SB and only 12% of them included an objective measure or some corroboration of the objective and subjective measure of SB.

A number of future recommendations should be considered with respect to the findings presented herein. There is a need for more longitudinal, prospective studies to be completed examining cognitive and motivational determinants of sedentary behavior. Only nine of the 25 reviewed studies were prospective in design and majority of these had relatively acute timelines (i.e., 7 to 14 day period). Studies that examine the association between cognitive and motivational factors and context-specific sedentary behavior over longer durations are required. The majority of the reviewed studies (i.e., 20 out of 25) employed solely self-reported estimates of sedentary behavior through a range of questionnaires, which differed in their outcomes assessed. Because of its high prevalence and habitual nature, sedentary behavior may be very diffıcult to recall accurately. It is recommended for future research in this field of inquiry to use accelerometers and/or inclinometers in conjunction with self-report methods. There was widespread variability between studies in the analytical methods used to identify correlates of sedentary behavior, as well as in the effect sizes reported. Consistent with the recommendations made by Rhodes et al. [17], researchers are encouraged to report standardized effect sizes along with the significance criterion when presenting their findings regarding cognitive and motivational factors related to sedentary behavior. This will allow for a meta-analysis to be conducted in this domain so the magnitude of cognitive and motivational constructs related to sedentary behavior can be evaluated and understood.

Replication of theory-based studies measuring sedentary-specific cognitive and motivational factors in high sedentary populations and contexts where sedentary behaviors are dominant is strongly recommended. These studies should also work on refining and validating instruments used to assess cognitions and conations (i.e., motivation) related to sedentarism. As noted in this review, a number of studies adapted physical activity scales or used non-validated tools to assess cognitive and motivational factors. The development of psychometrically validated tools and testing of theory is important for identifying and differentiating between protective and risk factors for sedentarism at varying life stages and across sedentary domains. This will allow researchers to identify the important cognitive and motivational correlates that should be targeted in interventions designed to reduce sedentary behavior. Owen and colleagues [5] suggested that the “primary strategic goal for research on sedentary behavior determinants and interventions is to integrate evidence to identify effective or promising strategies to reduce sitting time.” Further, Rhodes et al. [17] proposed that cognitive, social, and environmental correlates seem better suited for intervention efforts to reduce sedentary behavior. Theoretical behavior change models have been useful in identifying cognitive and motivational factors that have been shown to be associated with sedentary behavior, however, the manipulation of these variables for purposes of behavior change interventions to reduce sedentary behavior has yet to be extensively examined. For instance, Carr and colleagues [52] conducted a randomized controlled trial and demonstrated that an intervention grounded in Social Cognitive theory led to reduced sedentary time among middle-aged, sedentary and overweight adults working in sedentary jobs. In another successful study, Gardiner and colleagues [53] demonstrated that an intervention to reduce and break up sedentary time in older adults using Social Cognitive theory and behavior choice theory led to decreased sedentary time, increased breaks, and increased light-intensity physical activity and moderate-to-vigorous physical activity. While promising, further inquiry into the development of theory-based interventions targeting cognitive and motivational constructs with the goal of sedentary behavior reduction is needed.

Another potential theoretical model of interest for use in the sedentary behavior domain is the Health Action Process Approach [14] (HAPA). The HAPA model includes many variables that are similar to those shown in this review to be associated with sedentary behavior. This model holds several advantages over other models for intervention design and delivery in that it is a dynamic rather than static model. According to the HAPA model, successful behavior change involves both a pre-intentional motivational phase in which intention is formed and a post-intentional volitional phase in which intention is translated into action. To this end, the HAPA attempts to bridge the ‘intention–behavior gap’ inherent with other behavior change models (e.g., PMT, TPB) with action planning, coping planning, and action control components [54]. The HAPA model's effectiveness to explain the adoption and maintenance of numerous health behaviors has been demonstrated [14]. It is anticipated that the HAPA will also be of value in the sedentary behavior domain. It is recommended that the same line of inquiry be followed with HAPA as with previous behavior change models. First, valid and reliable HAPA sedentary constructs must be developed and then show an association to sedentary behavior. If relationships are found, the constructs must be targeted and modified through action and coping planning interventions with the goal of sedentary behavior reduction. Maher and Conroy [37], to our knowledge, are among the first to test a HAPA-based model of sedentary behavior and directly link planning, a key component of the HAPA model, with sedentary behavior. Maher and Conroy [37] highlighted that with other health behaviors, planning has been shown to be a crucial factor for bridging the goal intention-behavior gap. Their findings suggest that planning context-specific substitutes for sedentary behavior may be a promising approach for overcoming strong sedentary habits.

For purposes of this review, studies examining cognitive and motivational correlates of sedentary behavior from a qualitative approach were excluded. However, it is important to acknowledge that qualitative studies in this field of study exist and may potentially contribute to a deeper understanding of the role that cognitive and motivational factors play in sedentarism. For instance, Deliens, Deforche, De Bourdeaudhuij, and Clarys [55] used focus group discussions to examine a range of determinants of physical activity and sedentary behavior in university students, including perceived enjoyment, modeling, social support, and self-discipline. Similarly, this review was interested in the role of cognitive and motivational factors as determinants of sedentary behavior; as a result, studies examining affect (e.g., feelings, mood, stress, depression, coping behavior), physical self-perceptions (e.g., physical conditioning), health-related quality of life (e.g., physical function), and personality (e.g., traits, resilience) factors were excluded. It is recognized that these factors may also hold importance for a complete understanding of sedentary behavior determinants. For example, Uijtdewilligen, Singh, Chinapaw, Twisk, and van Mechelen [56] investigated the role of problem-focused coping, emotion-focused coping, and personality traits (i.e., inadequacy, social inadequacy, rigidity, self-esteem, self-sufficiency/recalcitrance, dominance, hostility) as person-related determinants of TV viewing and computer time in a cohort of young Dutch adults. They found that higher rigidity and self-sufficiency/recalcitrance were positively associated with TV time, whereas higher scores on self-esteem were significantly associated with higher computer time. Further, Breland, Fox, and Horowitz [57] examined the relationship between daily screen time and depression in a cross-sectional sample of overweight or obese minority women. Independent of physical activity, findings showed that engaging in high levels of daily screen time was associated with increased depression risk. These types of studies are warranted if we are to gain a more comprehensive understanding of the role psychological factors play in sedentarism.

In conclusion, a number of cognitive and motivational factors were identified that were associated with sedentarism. Among sedentary behavior-related cognitions, risk factors for greater sedentary time included having a more positive attitude towards sedentary behavior, perceiving greater social support/norms for sedentary behavior, reporting greater sedentary behavior habits, having greater intentions to be sedentary, and having higher intrinsic, introjected, and external motivation towards sedentary behavior. Protective factors associated with lower sedentary time included having greater feelings of self-efficacy/control over sedentary behavior and greater intentions to reduce sedentary behavior. Among physical activity-related cognitions, protective factors for lower sedentary behavior included a more positive attitude towards physical activity, having greater social support/norms for physical activity, greater self-efficacy/control for physical activity, higher physical activity intentions, and higher intrinsic and identified motivation towards physical activity. In addition, feeling more supported and empowered in general was related with lower levels of sedentary behavior. To further extend our understanding of the relation between cognitive and motivational factors and sedentary behavior, more longitudinal, theory-driven studies examining cognitions and motivation from a sedentary perspective are required.

**Table S1.** Studies examining social-cognitive and motivational determinants of sedentary behavior.

Study	Sample	Design	Determinants examined	Sedentary behavior measure	Data collection timeline	Results: Correlates/predictors of sedentary behavior
Atkin, Corder, Goodyer, et al., 2015	Convenience sample - N = 738 - Large sample of early adolescents, aged 14 years; schools located in the counties of Cambridgesh ire and Suffolk - United Kingdom (UK)	Cross-sectional	Non-theory driven Variables: - Adolescents perceived family functioning - Friendship quality	Direct: - Physical activity and sedentary time were assessed objectively using combined heart rate and movement sensing (Actiheart, CamNtech Ltd, Papworth, UK) - Participants asked to wear for remainder of the testing day and then for four consecutive days, including two weekend days Self-report: - Separately for week and weekend days, time spent per day in each of the following sedentary behaviors: watching TV (inc. video/DVD), using the internet, playing video games, doing homework, and reading for pleasure.	Single assessment	*Association of family functioning and friendship quality with sedentary time:* - Higher scores on the good friendship qualities subscale was associated with lower sedentary time on weekdays (−10.34; −17.03, −3.66). *Association of family functioning and friendship quality with self-reported sedentary behaviors:* - Boys from better functioning families were less likely to report playing video games at the weekend (OR; 95% confidence interval: 0.73; 0.57,0.93) or reading for pleasure (weekday: 0.73; 0.56,0.96 weekend: 0.75; 0.58,0.96). - Boys who attained higher scores on the good friendship qualities scale were less likely to play video games at the weekend (0.61; 0.44,0.86) or report high homework on weekdays (0.54; 0.31,0.94). - A higher score for good friendship qualities was associated with lower odds of girls playing video games during the week (0.76; 0.58,1.00) or reading for pleasure at the weekend (0.61; 0.42,0.88). Girls that reported fewer friendship difficulties had lower odds of high TV viewing (0.76; 0.62,0.93) or playing video games (0.71; 0.52,0.97) at the weekend, and lower odds of reading for pleasure (0.63; 0.49,0.81) or reporting high homework on weekdays (0.70; 0.52,0.95).
Bai, Chen, Vazou, et al., 2015	- N = 1,552 - Students in 3rd through 12th grade from 18 schools involved in PE4Life training programs (4 schools in Arkansas and 14 schools in Iowa) - 540, 318, and 694 youth from 8 elementary, 3 middle, and 7 high schools, respectively - Arkansas, USA; Iowa, USA	Cross-sectional	Psychological, theory-driven: youth physical activity promotion (YPAP) model Variables: - Children's attraction to PA - Perceived physical competence	Self-report: - The Youth Activity Profile (YAP): Based conceptually on the widely used Physical Activity Questionnaire - Online survey tool designed to assess youth participation in PA (at school and at home) as well as their SB - The first five items assess the PA level atschool (PAS) in various school time periods. The next five items measure PA level at home (PAH) in various time periods. The last five items measure SB including time spent watching TV, playing video games, on the computer, on the phone/texting, and overall SB.	Single Assessment	*Variables correlated with sedentary behavior:* - Psychosocial variables (i.e., attraction to PA and perceived competence) had low negative correlations with SB (r = –.19 to –.34, *p* < .05) - Elementary school: Attraction to PA (r = –.29); Perceived competence (r = –.19) - Middle school: Attraction to PA (r = –.34); Perceived competence (r = –.33) - High school: Attraction to PA (r = –.33); Perceived competence (r = –.23) *Variables predicting SB:* - Perceived Competence significantly predicted SB ( *β* = –.28; 95% CI: –0.22, –0.14). Attraction to PA statistically significantly predicted SB in all age groups ( *β* = –.49; 95% CI: –0.22, –0.14). Thus, the students who felt more competent in PA and attracted to PA were more likely to be active and less sedentary. The effect of Perceived competence on SB was reduced but remained statistically significant after controlling for the effects of attraction to PA. Bootstrapping mediation analysis confirmed that perceived competence had a statistically significant indirect effect on SB (IE = .13, *p* < .05).
Busschaert, De Bourdeaudhu ij, Van Cauwenberg, et al., 2016	Random sample - N = 188 - Adult inhabitants of the city of Sint-Niklaas, aged 25–60 years - Sint-Niklaas, Belgium	Longitudinal prospective design	Non-theory driven: Intrapersonal, social-cognitive and physical environmental variables Variables: Intrapersonal: - BMI, occupational status, residential area, depressive symptoms, children living at home, family situation, occupational classification, educational level and sex Social-cognitive: - attitude, self-efficacy, norm, social norm, social support and modelling Physical environmental: - TV set, other TV viewing equipment - computer equipment, other equipment for computer use - number of operational motorized vehicles - occupational desks at work or not	Self-report: Context-specific sitting time (i.e. TV-viewing, computer use, motorized transport and occupational sitting) - 11 items targeting sitting behavior in the past 7 days	One-year (April 2013–April 2014) Baseline: All variables One-year follow-up: All variables	*Social-cognitive correlates of TV-viewing, computer use, motorized transport and occupational sitting at baseline:* - A one-unit higher score for ‘I enjoy watching TV for many hours’ (attitude 3) and ‘I find TV a way to relax’ (attitude 4) was associated with respectively 19 and 12% more sitting while watching TV. Also, a one-unit higher score for ‘time partner spend watching TV’ (modelling 1) was associated with 5 % more sitting while watching TV. - A one-unit higher score for ‘I think using a computer is pleasant’ (attitude 1), ‘I enjoy using a computer for many hours’ (attitude 3) and ‘I think that I spend too much timeon the computer’ (norm) was associated with respectively 34, 17 and 24 % more sitting while using a computer. A one-unit higher score for ‘I consider it possible that I do not use a computer for some days in the week’ (self-efficacy 1) was associated with 13 % less sitting while using a computer. - A one-unit higher score for ‘I think that I spend too much time using motorized transport’ (norm) was associated with 14 % more sitting during motorized transport. A one-unit higher score for ‘I consider it possible to take the bicycle or to go by foot spontaneously even if it is possible to use a car’ (self-efficacy 3) was associated with19 % less sitting during motorized transport. *Relationship between changes in social-cognitive predictors from baseline to follow-up and changes in TV-viewing, computer use, motorized transport and occupational sitting:* - An increase from baseline to follow-up with one unit on the five-point Likert scale for ‘I enjoy watching TV for many hours at a time’ (attitude 3) was associated with 7.96 min/day more sitting while watching TV at follow-up. An increase from baseline to follow-up with one unit on the eight-point Likert scale for ‘time partner spend watching TV’ (modelling 1) was associated with 9.91 min/day more sitting while watching TV at follow-up. - An increase from baseline to follow-up with one unit on the five-point Likert scale for ‘I consider it possible to park the car somewhat further spontaneously and to walk the remaining distance’ (self-efficacy 2) was associated with 8.48 min/day more sitting during motorized transport at follow-up. More active transport to go to work/school (modelling 1) from baseline to follow-up of the partner was associated with 16.47 min/day more sitting during motorized transport at follow-up of the respondent.
Chang & Sok (2015)	Convenience sample - N = 306 - Elderly persons with hypertension (HTN) who were registered at three public health centers of three boroughs in Seoul, Korea - Seoul, Korea	Cross-sectional	Theory-driven: Empowerment theory Psychosocial variables: - Self-efficacy for PA - Social support for PA - Empowerment - Depressive symptoms Other variables: - Demographic characteristics - Disease related characteristics (e.g., perceived health) - Behavioral characteristics (e.g., alcohol consumption, PA)	Self-report: - International Physical Activity Questionnaire -Short Form(IPAQ-SF) - Single question about sitting from the IPAQ-SF - Time in sedentary behavior was assessed in minutes over the previous 1 week, including time spent sitting at work, at home, in class, and during leisure activities as well as sitting or lying time spent at a desk, meeting friends, reading books, moving in a car, and watching TV.	Single assessment	*Characteristics related to sedentary behavior:* - A higher number of minutes of sedentary behavior were associated with lower levels of empowerment ( *r* = –.498, *p* < .001) and self-efficacy for PA ( *r* = –.297, *p* < .001) *Predictors of sedentary behavior:* - Empowerment was found to be the strongest predictor of a high level of sedentary behavior ( *β* = –.394, *p* < .001).
Conroy, Maher, Elavsky, et al., 2013	Convenience sample - N = 128 (53 men and 75 women with a mean age of 21.3 years ( *SD* = 1.1)) - College students, recruited from advanced undergraduat e courses - PA, USA	Prospective study	Psychological, theory-driven: Dual-process theory of motivation Variables: - Intentions to limit sedentary behavior - Sedentary behavior habits	Direct: - ActiGraph GT3X accelerometer (ActiGraph, Pensacola, FL) Self-report: - International Physical Activity Questionnaire (IPAQ) - Four-item measure included questions about the duration of time spent engaged in vigorous physical activity, moderate physical activity, walking, and sitting that day - Sedentary behavior scores were expressed as the number of minutes that a participant spent sitting each day	14-day ecological momentary assessment study; daily sampling schedule	*Variables correlated with sedentary behavior:* - Habit strength for sedentary behavior was positively associated with sedentary behavior ( *r*s = .20, .36) and unassociated with physical activity ( *r*s = −.03, −.06). People with stronger sedentary habits reported, on average, weaker intentions to limit their sedentary behavior ( *r =* −.25). Intentions to limit sedentary behavior were associated with less sedentary behavior ( *r*s ranged from –.23 to –.56) and more physical activity ( *r*s ranged from .18 to .30). Sedentary behavior and physical activity exhibited moderate to strong negative correlations ( *r*s ranged from –.22 to –.59). - Self-reported SB: Daily deviations in intentions were significantly associated with decreased self-reported sitting time (γ _100_ = –0.09, *p* < .001; i.e., people who reported stronger intentions to limit their sitting time subsequently reported sitting less) - Both the overall strength of intentions to limit sitting time (γ _02_ = –0.22, *p* < .001) and sedentary habit strength (γ _03_ = 2.13, *p*< .001) were significantly associated with self-reported sitting time (in opposite directions as expected) - Directly-monitored SB: Daily deviations in intentions to limit sedentary behavior were associated with decreased sedentary behavior (γ _100_ = –1.40, *p* = .003) - Habit strength was associated with greater sedentary behavior (γ _03_ = 23.97, *p* = .04 - Sedentary behavior also varied within people as a function of concurrent physical activity, the day of week, and the day in the sequence of the monitoring period.
De Cocker, Duncan, Short, et al., 2014	Random sample - N = 993 - Employed Australian adults - Australia	Cross-sectional	Non-theory driven: Socio-demographic, health-related, work-related, and psychosocial factors Variables: - Socio-demographic (country of birth, gender, age, education, income) - Health-related (general health, weight, BMI, physical activity) - Work-related (employment status, occupational task, occupational classification) - Sedentary-specific psychosocial (Social norm towards sitting less at work; Social support to sit less at work; Self-efficacy: sit less the next month at work; Self-efficacy: certainty to sit less at work; Control to sit less; Advantages of sitting less at work; Disadvantages of sitting less at work; Intention to sit less at work)	Self-report: Occupational sitting time: - Workforce Sitting Questionnaire (WSQ) - Assesses time spent sitting on a workday and a non-workday for the last seven days while (1) travelling to and from places; (2) at work; (3) watching TV; (4) using a computer at home; and (5) doing other leisure activities. - Time spent sitting at work was computed as follows: [(average daily sitting-time at work on workdays × number of workdays) + (average daily sitting-time at work on non-workdays × number of non-workdays) / 7] to get the average daily occupational sitting-time.	Single assessment	*Differences in occupational sitting-time between psychosocial categories:* - Participants with higher social norms and less control to reduce sitting, those finding it valuable, pleasant, healthy, relaxing (all *p* < 0.001) to sit less, those disagreeing that sitting less is not beneficial at all ( *p* = 0.001), those disagreeing that sitting less is aggravating health problems ( *p* = 0.041), and those intending to sit less ( *p* < 0.001) reported higher occupational sitting-time compared to the respective comparison categories *Associations of psychosocial correlates with occupational sitting-time:* - Univariate regressions: Social norm towards sitting less at work ( *β* = 45.8), self-efficacy: certainty to sit less at work ( *β* = 0.4), control to sit less ( *β* = 14.6), advantages of sitting less at work ( *β* = 46.5), disadvantages of sitting less at work ( *β* = –34.6), intention to sit less at work ( *β* = 71.8) - The full multiple regression model showed that, of the eight psychosocial factors, only higher awareness of advantages of sitting less at work was associated with more occupational sitting time ( *β* = 0.673; 95% CI: 0.06–1.28; *p* = 0.030). - Employment status and occupational classification moderated the association between control to sit less and occupational sitting. A lack of control to sit less was associated with higher occupational sitting in part-time and full-time workers, but not in casual workers; and in white-collar and professional workers, but not in blue-collar workers.
Gaston, De Jesus, Markland, et al., 2016	Convenience sample - N = 571 individuals (416 females and 155 males; M _age_ = 23.93 years, SD = 6.18, Range = 18–54 years) - University students or staff - Ontario, Canada	Cross-sectional - An internal computer-generated randomization scheme (via Survey Monkey) directed participants to one of five groups: general, weekday work/school, weekday leisure/recreation, weekend work/school, and weekend leisure/recreati on. - Depending on group assignment, the sedentary-derived motivation items were preceded by a different introduction.	Theoretical Model: organismic integration theory (OIT), a sub-theory of self-determination theory (SDT) Variables: - Motivation type(s): - External - Introjected - Identified - Intrinsic	Self-report: Sedentary Behavior Questionnaire (SBQ) - 12-item modified version - Completed twice: once referring to an average weekday and once referring to an average weekend. - The SBQ included both work/school and leisure/recreat ion activities. - Five separate sedentary behavior time scores were computed, an overall score (i.e., average time spent per day in sedentary activity) as well as time spent in leisure/recreat ional and work/school activities on weekdays and weekends, separately.	Single assessment	*Pearson correlations for sedentary behavior and regulation type:* - Weekend work/school: external regulation ( *r* = .18, *p* < .05), intrinsic motivation ( *r* = –.27, *p* < .001) - Weekday work/school: introjected regulation ( *r* = .22, *p* < .05) - Weekday leisure/recreation: intrinsic motivation ( *r* = .19, *p* < .050) - Weekend leisure/recreation: intrinsic motivation ( *r* = .31, *p* < .001) - There were no significant relations between identified regulation and behavior. *Variables predicting sedentary behavior:* - Weekend work/school: external regulation, intrinsic motivation - Weekday work/school: introjected regulation - Weekday leisure/recreation: intrinsic motivation - Weekend leisure/recreation: intrinsic motivation - The percent of variance explained ranged from 3% (weekday leisure/recreation) to 10% (weekend work/school).
Gebremariam, Totland, Andersen, et al., 2012	- N = 885 - Group of Norwegian children in the transition between childhood and adolescence. - Students from 25 control schools of an intervention study, the HEalth In Adolescents (HEIA) study. - Average age at baseline = 11.2, standarddeviation ± 0.3) - Norway	Longitudinal prospective study	Non-theory driven Variables: - Perceived parental regulation - Self-efficacy related to barriers for PA - BMI - Pubertal development category - Ethnicity - Living status of children (i.e., those living with married or cohabitating parents; those living with their father or mother alone, equally with their mother or father, grandparents or another adult) - Parental education	Self-report: - TV/DVD use, computer/elec tronic game use and total screen time (TST; hours/week) - Four questions with pre-coded answer categories assessing screen-based sedentary behaviors on weekdays and weekends - The answer categories for TV/DVD use were: half hour [0.5], one hour [1], two hours [2], three hours [3], fourhours [4], five hours or more [5]. - The answer categories for computer/elec tronic game use were: no playing [0], half hour or less [0.5], one hour [1], two hours [2], three hours [3], four hours or more [4]. - TSTcomputed	Baseline: September 2007 1 ^st^ follow-up: May 2008 2 ^nd^ follow-up: May 2009	*Factors associated with an increase in TST between BL and T2:* - Among males, self-efficacy related to barriers to PA (B = −2.16 (−3.60, −0.73)) was inversely related to an increase in TST, indicating a decrease of around 2.2 hours per week per unit increase in self-efficacy score. *Predictors of tracking of high TST:* - Results of the multinomial regression analysis show that, among girls, children with low self-efficacy related to barriers to PA were more likely to track high TST (OR = 2.30, C.I. = 1.13-4.69, *p* < .05) compared to children with high self-efficacy. - Among males, boys with low self-efficacy related to barriers to PA were also more likely to track high TST (OR = 6.83, CI = 3.22-14.45, *p* < .001) than the group with high self-efficacy.
Ham, Sung, & Kim, 2013	Convenience sample - N = 370 - School-age children - South Korea	Cross-sectional	Non-theory driven: sociodemographic, psychosocial, and behavioral characteristics Variables: - General and family characteristics - Sleep duration - Stress - Pros and cons of exercise - Exercise self-efficacy - Eating behaviors	Self-report: - Screen time - A single question was used for the determination of screen time, “how many hours per day have you spent viewing TV/video, using computers, and playing video games during the past month?” - Scored on a nominal scale (1 = less than 1 hr, 2 = 1–2.9 hr, 3 = 3 or more hr).	Single assessment	*Differences in Psychosocial Characteristics According to Screen Time:* - Increased screen time showed a significant association with pros and cons of exercise and exercise self-efficacy ( *p* < .05). Those with screen time of 3 or more hr/day had lower pros of exercise ( *F* = 3.537, *p* = .030), higher cons of exercise ( *F* = 6.829, *p* = .001), and lower exercise self-efficacy ( *F* = 3.354, *p* = .036), compared to their counterparts. *Multinomial Logistic Regression Analysis of Factors Associated With Screen Time:* - Pros and cons of exercise, and self-efficacy did not show a significant association with screen time among subjects with screen time between 1 and 2.9 hr/day. - Among subjects with screen time of 3 or more hr/day, cons of exercise (OR = 2.844, 95% CI = [1.285, 6.298]) showed a significant association with screen time. Other variables including pros of exercise and self-efficacy did not show a significant association with a screen time among subjects with screen time of 3 or more hr/day.
He, Piché, Beynon, et al., 2010	Random sample - N = 508 student-parent pairs - Elementary school students and their parents (i.e., grades 5 and 6 students) - London, Ontario, Canada	Cross-sectional - Children were categorized into 2 groups: “low-screen users,” who met the CPS guidelines, and “high-screen users,” who exceeded Canadian Pediatrics Society (CPS) guidelines.	Psychological, theory-driven: Social-ecological model; Attitude-Social Influence-Self-efficacy Model (ASE) Variables: - Attitude (i.e., how they felt about excessive screen use and what motivates them to use screens) - Social influence (i.e., perceptions of parental expectations and controls over screen use) - Intention	Self-report: Children's screen-related behaviors - Brief self-administered questionnaire, The Child Sedentary Activity Questionnaire (CSAQ), - Designed to measure children's re-call of hours spent each day of the previous week watching television or videos and playing computer and video games outside of school hours. - Children's school screen time was estimated by asking grade 5 and 6 classroom teachers about the number of hours their students spent watching television and videos or using computers in the classroom each day. - Total screen time was the combined amount of screen-related activities during in-school and out-of-school hours.	Single assessment	*Differences in variables btw low- and high-screen users:* - A significantly smaller proportion of high-screen users held negative attitudes about screen use ( *P* < .01) - Intentions: More than two thirds of children indicated that they would elect to spend more time engaged in physical activities if they were “given the choice”; however, fewer high-screen users than low-screen users ( *P* < .01) chose to do so. - Significantly fewer high-screen users had perceived parental limits on TV ( *P* < .05), video games ( *P* < .01), or the computer for nonhomework use ( *P* < .01) on weekends.
Hoyos Cillero, Jago, & Sebire, 2011	- n = 247 primary school-aged and n = 256 secondary school-aged children - Spanish school children - Spain	Cross-sectional	Psychological, theory-driven: Social cognitive theory Variables: - Individual factors (self-efficacy to reduce screen-viewing time, behavioral capability) - Social factors (sedentary group norms, social reasons for sedentary behaviors, perceived maternal rules for screen-viewing)	Self-report: Screen-viewing - Self-administered questionnaire comprising six items assessing hours of TV viewing, computer playing and console playing for an average weekday and weekend day. - Daily TV, computer and console games-playing times were summed to create an overall screen-viewing variable. - In addition, children were classified as not meeting TV and overall screen-viewing guidelines in accordance with AAP guidelines (≥2 h/day).	Single assessment	*Relationship between screen-viewing behaviours and variables:* - Stronger sedentary group norms (OR 1.26 [1.04–1.53], *p* = 0.017) and higher behavioural capability (OR 1.25 [1.01–1.54], *p* = 0.036) were associated with watching TV ≥2 h/day on weekdays and weekends respectively for primary school-aged females. - For younger males, having lower paternal rules (for weekdays OR 0.83 [0.75–0.90], *p* < 0.001; and for weekends OR 0.68 [0.50–0.93], *p* = 0.016) was a significant predictor for exceeding TV viewing guidelines. - For older females, having stronger sedentary group norms (OR 1.36 [1.17–1.58], *p* < 0.001) was associated with increased likelihood of exceeding TVviewing guidelines on weekdays and weekends respectively. - The significant predictors for younger females playing console games ≥2 h/day on weekdays were higher maternal rules (OR 1.88 [1.30–2.70], *p* = 0.001) and lower paternal rules (OR 0.49 [0.30–0.79], *p* = 0.004) on weekdays. On weekends, lower self-efficacy (OR 0.61 [0.37– 0.99], *p* = 0.047) was also a strong determinant for this subgroup. - For younger males, having stronger sedentary group norms (OR 1.28 [1.05–1.57], *p* = 0.013), stronger social reasons for engaging in screen-viewing (OR 1.24 [1.00–1.53], *p* = 0.048) and lower maternal rules (OR 0.57 [0.33–0.97], *p* = 0.039) were significant determinants for console games-playing ≥2 h/day on weekdays. On weekends, higher behavioural capability (OR 1.37 [1.09–1.72], *p* = 0.006) and lower maternal rules (OR 0.78 [0.64–0.94], *p* = 0.012) were also significant predictors for this subgroup. - Older females having lower paternal rules (OR 0.57 [0.45–0.70], *p* < 0.001) were more likely to engage ≥2 h/day in console games-playing on weekdays and on weekends respectively. - For older males, having stronger sedentary group-norms (OR 1.22 [1.00–1.50], *p* = 0.047) was associated withplaying console games ≥2 h/day on weekends. - For younger females, stronger sedentary group norms (OR 1.19 [1.02–1.40], *p* = 0.027) and lower paternal rules (OR 0.70 [0.50–0.98], *p* = 0.043) were significant predictors for exceeding screen-viewing guidelines on weekdays. On weekends, higher behavioural capability (OR 1.30 [1.09–1.56], *p*= 0.003) was also a strong predictor for this subgroup. - Lower paternal rules (for weekdays OR 0.90 [0.82–0.99], *p* = 0.046 and for weekends OR 0.64 [0.45–0.90], *p* = 0.011) was a significant predictor for younger males exceeding screen-viewing guidelines. On weekends, higher behavioural capability (OR 1.37 [1.13–1.65], *p*= 0.001) was also a strong predictor for this subgroup. - Older females with strong sedentary group norms (OR 1.34 [1.01–1.77], *p* = 0.039) were more likely to spend ≥2 h/day engaged in overall screen-viewing time on weekdays. Lower self-efficacy (OR 0.10 [0.02–0.47], *p* = 0.003), higher maternal rules (OR 4.16 [1.50–11.5], *p* = 0.006) but lower paternal rules (OR 0.17 [0.07–0.44], *p* < 0.001) were also significant determinants for exceeding screen-viewing guidelines on weekends for this subgroup. - For older males, lower paternal rules (OR 0.76 [0.60–0.97], *p* = 0.027) was a significant predictor for exceeding screen-viewing guidelines on weekends.
Huang, Wong, & Salmon, 2013	Random sample - N = 303 - School children in grades 4–6 recruited from 16 primary schools - Hong Kong, China	Cross-sectional	Non-theory driven: Demographic information, individual, social, environmental variables Variables: - Sex of child - Parent's education level - Children's BMI - Children's self-efficacy for PA - Child self-reported number of siblings at home - Child's perceived family and peer support - Perceived parental enjoyment of SBBs - Parental role modeling - Guidance/Rules on SBBs - The home environment - Perceived neighborhood safety - Social environment in neighborhood - Sports facilities in neighborhood	Self-report: Physical activity and screen-based behaviors (SBBs; i.e., TV viewing, electronic games playing, and Internet use) - Children's Leisure Activities Study Survey questionnaire -Chinese version(CLASS-C) - Children reported the total time they spent in a checklist of 31 physical activities and SBBs during past week - Scored by calculating daily minutes spent in MVPA and SBBs	Single assessment	- Less family support for PA ( *β* = –0.73; 95% CI: –1.34, –0.13) was associated with higher TV viewing time in the crude model among boys ( *p* < 0.05) - In the hierarchical model, family support for PA ( *β* = –0.54; 95% CI: –1.10, 0.00) was negatively associated with boys' TV viewing time ( *p* < 0.05) - Self-efficacy ( *β* = –0.77; 95% CI: –1.69, 0.15; *p* < 0.1) and family support for PA ( *β* = –1.03; 95% CI: –1.55, –0.51; *p* < 0.01) were associated with boys' internet use/e-games playing - Self-efficacy ( *β* = 1.15; 95% CI: 0.24, 2.06; *p* < 0.05) and peer support for PA ( *β* = 0.91; 95% CI: –0.10, 1.92; *p* < 0.1) were correlated with girls' internet use/e-games playing - In the full model for boys, family support for PA ( *β* = –0.86; 95% CI: –1.41, –0.30) was negatively associated with Internet use and e-games playing ( *p* < 0.01). - Interestingly, girls with higher self-efficacy for PA ( *β* = 1.06; 95% CI: 0.02, 2.11) reported more time spent using the internet and playing e-games ( *p* < 0.05)
Janssen, Basterfield, Parkinson, et al., 2015	Representative sample - N = 365 - Children and adolescents; 9.3 (±0.4) years at baseline and 12.5 (±0.3) years at follow-up. - Northeast England, UK	Longitudinal prospective study	Theory-driven: Socio-ecological model - 20 measures of potential determinants of changes in both sedentary time and fragmentation between 9 y and 12 y Variables: - Demographic and biological domain (gender; age; BMI; socioeconomic status (SES); maternal age; maternal BMI; parent outside of family home) - Psychological domain (interest in sedentary behaviors) - Behavioral domain (time spent on electronic devices; change in time spent in objectively measured moderate-to-vigorous intensity physical activity (MVPA); attendance at sports clubs) - Socio-cultural environmental domain (parenting rules in relation to sedentary behavior/screen time; parental modelling of sedentary behavior/screen time; parent enjoyment of sedentary behavior/screen time; parent daily sedentary behavior/screen time) - Physical environmental domain (number of TVs in the home; TV in bedroom; computer at home; subscription-based television services available; seasonality)	Direct: Sedentary time and sedentary fragmentation: - ActiGraph accelerometry - In brief, participants were asked to wear the ActiGraph GT1M (ActiGraph Corporation; Pensacola USA) on a waist belt duringwaking hours for 7 days - Sedentary time was expressed in absolute terms (minutes per day) when describing the magnitude of daily sedentary, but in the analyses was expressed as a % of wear time to minimize variation in sedentary time due to wear time. - Sedentary fragmentation was expressed using the fragmentation index - A greater fragmentation index indicates that time spent sedentary is more fragmented (interrupted).	Three-year follow-up Baseline: September 2008 to August 2009 Follow-up: January 2012 to November 2012 - Baseline measures were taken when children were 8–9 y of age (from here on referred to as 9 y) and when children were 11–12 y (from here on referred to as 12 y).	*Univariate analyses of determinants associated with change in sitting time:* - Child interest in sedentary behavior ( *β* = 1.12; 95% CI: – 0.20–2.41) - More interest was associated with greater increase in sedentary time.
Kremers & Brug, 2008	- N = 383 - Adolescents (mean age = 13.5, *SD* = 0.6; range 12–17 y; 55.4% girls) at five schools in the region around the town of Nijmegen, The Netherlands - The Netherlands	Cross-sectional	Non-theory driven Variables: - Self-report habit index (SRHI; habit strength for watching TV and using a computer) - Pros of watching TV and using a computer - Cons of watching TV and using a computer - Intention for SB	Self-report: television viewing and using a computer - Frequency measure with respect to these behaviors consisted of six items, assessing the number of minutes that the respondents spent on these behaviors. - Two items assessed the number of days they engaged in watching TV or video and using a computer (surfing the Internet, playing games, chatting) during a normal week. Four additional items assessed the amount of time that the adolescents engaged in each of these behaviors during a regular weekday (two items) and during a regular weekend day (two items). - A sum score was computed of the total number of minutes spent per day watching TV or using a computer.	Single assessment	*Correlations Between Pros, Cons, Habit Strength and Behavioral Measure of Sedentary Behavior Among Adolescents:* - The SRHI score correlated positively with the behavioral measure ( *r* = 0.50, *p* < .001), intention ( *r* = 0.37, *p* < .001), and the perceived pros ( *r* = 0.56, *p* < .001) and correlated negatively with the cons ( *r* = –0.21, *p* < .001) - Sedentary intentions correlated positively with sedentary behavior ( *r* = 0.29, *p* < .001) - Perceived pros correlated with sedentary behavior ( *r* = 0.37, *p* < .001) - Perceived cons correlated negatively with sedentary behavior ( *r* = –0.29, *p* < .001) *Hierarchical Multiple Regressions to Test Moderating Influence of Habit on the Pros–Intention, Cons–Intention and Intention–Behavior Relationship:* - Hierarchical-regression analyses with intention as the dependent variable revealed main effects of habit and perceived pros, as well as a significant habit x pros interaction. Simple slope analyses indicated a significant relation between pros and intention in the weak-habit group ( *β* = 0.34; *t*[379] = 4.80; *p* < .001) and a nonsignificant relation ( *β* = 0.12; *t*[379] = 1.69) in the strong-habit group. The habit x cons interaction was not statistically significant. - Regarding the intention–behavior relationship, hierarchical regression revealed main effects for both intention and habit, as well as a significant habit x intention interaction. Simple slope analyses showed a significant relation between intention and behavior in the weak habit group ( *β* = 0.30; *t*[379] = 4.26; *p* < .001) and a nonsignificant association in the strong-habit group ( *β* = 0.08; *t*[379] = 1.21).
Lowe, Danielson, Beaumont, et al., 2015	Convenience sample - N = 31 - Advanced cancer patients diagnosed with brain metastases, aged 18 years or older, cognitively intact, and with palliative performance scale greater than 30%, were recruited from a Rapid Access Palliative Radiotherapy Program multidisciplin ary brain metastases clinic. - Cross Cancer Institute, Edmonton, AB, Canada	Prospective study	Theoretical Model: Theory of Planned Behaviour (TPB) Variables: - Attitudes to perform regular physical activity: affective and instrumental attitudes - Subjective norms (SN) - Perceived behavioral control (PBC) and self-efficacy for physical activity - Intention with respect to regularly being physically active	Direct: - activPAL™ accelerometer for 7 days (PAL Technologies Ltd, Glasgow, United Kingdom)	Single assessment - TPB variables: cross-sectional survey via face-to-face interviews to all participants - Participants asked to wear an activPAL™ accelerometer for up to 7 days	*TPB variables correlated with objectively measured sedentary behavior:* - Correlates of median time spent supine or sitting in hours per day were instrumental attitude (i.e., perceived benefits) of physical activity ( *r* = –0.42; *p* = 0.030) and affective attitude (i.e., perceived enjoyment) of physical activity ( *r* = –0.43; *p* = 0.024). - Correlation between intention and objectively measured sedentary behavior ( *r* = –0.32, *p* = 0.10) was not statistically significant, but potentially meaningful. *Differences in TPB variables between participants based on the median of 20.7 h spent sitting or supine per day:* - Participants who sat or were supine for greater than 20.7 h per day reported significantly lower instrumental attitude ( *M* = 0.7; 95% CI = 0.0–1.4; *p* = 0.051) and affective attitude ( *M* = 0.7; 95% CI = 0.0–1.4; *p* = 0.041) *Differences in objectively measured sedentary levels based on medical and demographic factors:* - Participants who were <60 years of age ( *M* = 19.4, 95% CI – 4.0–0.0, *p* = 0.055) recorded less time spent sit-ting or supine per day
Maher & Conroy, 2015	- N = 188 (89 female, 95 male, three did not report) - Undergraduat e students - USA	Prospective Experimental (7-day action planning intervention) - Before data collection, participants were assigned to one of four conditions in a 2 × 2 factorial design. The two experimental factors represented whether participants created or did not create a detailed plan describing when, where, and how participants would engage in physical activity the following day (Factor 1), or when, where, and how participants would limit or interrupt an extended period sitting the following day (Factor 2).	Psychological, theory-driven: Dual-process theories of health behavior motivation Variables: - Demographics - Habit strength (both PA and sedentary behavior habit strength) - Intentions to engage in PA - Intentions to reduce sedentary behavior	Self-report: Daily physical activity and sedentary behavior - International Physical Activity Questionnaire(IPAQ) - Adapted to focus on daily instead of weekly PA and SB - Asked to report the amount of time that they spent in physical activities for at least 10 min at a time that day as well as the total amount of time spent sitting that day	7-day protocol - Baseline + for the next 7 days, participants received an e-mail each night at 7:00 p.m. containing a link to access the questionnaire that included questions about their behavior that day and intentions for physical activity and sedentary behavior the following day and the planning intervention(s) correspondin g to their randomly assigned experimental condition.	- Sedentary behavior had positive weak correlations with sedentary behavior habit strength ( *r* = .17) but a negative medium-sized correlation with SB intentions (between-person *r* = –.33, within-person *r* = –.36). - The daily planning intervention to limit sedentary behavior (γ _01_, γ _02_, γ _03_) was not significantly associated with daily sedentary behavior. - Habit strength was a significant, positive predictor of sedentary behavior (γ _03_), so that people with stronger habits for sedentary behavior engaged in more sedentary behavior. - The interaction between daily planning and sedentary behavior habit strength was not a significant predictor of daily sedentary behavior (γ _05_). - Participants who had stronger usual intentions to limit or interrupt sedentary behavior had lower usual levels of physical activity (γ _06_). - On days when participants intended to limit or interrupt sitting time more than was typical for them, they reported lower levels of sedentary behavior (γ _10_).
Maher & Conroy, 2016	- N = 100 (n=67 women, n=33 men) - Community-dwelling older adults - USA	Prospective study	Psychological, theory-driven: Dual-process theory of motivation; habit model; The Health Action Process Approach (HAPA) Variables: - Intentions to limit SB - Task self-efficacy to limit SB - Outcome expectations for light-intensity PA - Risk perceptions - Sedentary behavior habit strength - Physical activity (i.e., IPAQ) - Physical symptoms - Temporal processes	Self-report: Daily self-reported sedentary behavior - 9-item scale which featured domain-specific sedentary activities included in other validated measures of older adults' sedentary behavior (i.e., watching TV, using computer, reading, socializing with friends, in transit, completing hobbies, etc.) Direct: - Objectively measured sedentary behavior - ActivPAL3 activity monitors used	14-day ecological momentary assessment study - Over the 14 days participants completed questionnaire s on their tablet at the beginning (measures included daily task self-efficacy, intentions, planning to limit sedentary behavior, sleep/wake times) and end of each day (measures included domain-specific sedentary time, physical activity, physical symptoms) and wore the activity monitor on their thigh during all sleeping and waking hours.	*Between- and within-person correlations between sedentary behavior (self-reported and objectively measured) and dual-process constructs:* - Self-reported and objectively measured sedentary behavior were moderately correlated ( *r*s = .38, .28). - Sedentary behavior (self-reported and objectively measured) had weak-to-moderate positive correlations with habit strength ( *r*s = .22, .18) and weak-to-moderate negative correlations with planning ( *r*s = –.10, –.21). - Planning had moderate positive correlations with intentions ( *r*s = .51, .58). - Intentions had strong positive correlations with task self-efficacy ( *r*s = .83, .83). - Intentions also had weak-to-moderate positive correlations with sedentary behavior risk perceptions and light-intensity physical activity outcome expectations ( *r*s = .20, .06, respectively) at the between-person level. - Intraclass correlation coefficients (ICC) were calculated to describe the proportion of variance in each variable attributable to between-person differences. ICCs indicated that approximately half of the variance in self-reported and objectively measured sedentary behavior and two thirds of the variance in task self-efficacy, intentions, and planning was the between-person variance, with the remainder driven by within-person factors and measurement error. *Multilevel model of daily sedentary behavior:* - Multilevel models predicting behavior revealed that sedentary behavior was (a) negatively associated with planning to reduce sedentary behavior at the within-person, and (b) positively associated with sedentary behavior habit strength (monitored behavior: γ _02_ = 19.97, *p* = .04). - There were no differences in objectively monitored sedentary behavior between participants who tended to form stronger or weaker plans (γ _01_ = –0.41, *p* = .24) but, as hypothesized, participants were less sedentary on days when they formed stronger-than-usual plans to limitsedentary behavior (γ _10_ = –0.51, *p* = .005). - As indicated by the pseudo- *R* ^2^, this model accounted for 14% of the variance in objectively measured sedentary behavior, with habit strength accounting for 9% and daily planning accounting for 5% of the explained variance. *Multilevel model of daily plans to limit SB:* - Plans to limit sedentary behavior were (a) positively associated with task self-efficacy at the within-person level (γ _10_ = 0.14, *p* = .001), but (b) negatively associated at the between-person level (γ _01_ = –0.59, *p* = .04), and (c) positively associated with intentions at the between- (γ _02_ = 1.17, *p* = .001) and within-person level (γ _20_ = 0.20, *p* = .004). - As indicated by the pseudo- *R* ^2^, this model accounted for approximately 20% of the variance in daily plans to limit sedentary behavior. Daily intentions accounted for 23%, daily task self-efficacy accounted for 10%, and usual intentions and task self-efficacy each accounted for 2% of the explained variance. *Multilevel model of intentions to limit SB:* - Intentions to limit sedentary behavior were (a) positively associated with task self-efficacy at the between (γ _01_ = 0.96, *p* = .001) and within-person level (γ _10_ = 0.61, *p* = .001), but (b) not associated with light-intensity physical activity outcome expectations, sedentary behavior risk perceptions, or sedentary behavior habit strength. - As indicated by the pseudo- *R* ^2^, this model accounted for approximately 44% of the variance in daily intentions to limit sedentary behavior, with daily task self-efficacy accounting for 80% and usual task self-efficacy accounting for 4% of the explained variance.
Norman, Schmid, Sallis, et al., 2005	Convenience sample - N = 878 - Ethnically diverse clinic-based sample of adolescentswho were 11 to 15 years old - San Diego County, California, USA	Cross-sectional	Theory-driven: Psychosocial and environmental variables - Psychosocial constructs assessed based on social cognitive theory, the transtheoretical model - Environmental variables derived from ecological models. Variables: Psychosocial: - behavior change strategies - pros and cons of change - self-efficacy - family support - enjoyment of sedentary behaviors - TV and video household rules - parent-reported support for PA Environmental: - home environment - neighborhood environment variables	Self-report: - Survey adapted from Robinson. - Participants were asked how much time they spent doing the following leisure-time sedentary behaviors: - watching TV (including videos on VCR/DVD); - playing computer or video games (such as Nintendo or Sega); - sitting and listening to music on the radio, audiotapes, or CDs; - sitting and talking on the telephone. - Questions were asked first for “most recent day when you were not in school” and then for the “most recent school day.” - An index of sedentary-behavior time was computed by summing the 4 items for non-school days.	Single assessment	*Associations between predictor variables and leisure-time sedentary behavior:* - Girls: Higher scores on change strategies (OR: 0.59; 95% CI: 0.45–0.76), pros (OR: 0.62; 95% CI: 0.51–0.77), and self-efficacy (OR: 0.45; 95% CI: 0.35–0.59) were related to decreased likelihood of being in the high-sedentary-behavior group. - Girls: High scores on cons (OR: 1.90; 95% CI: 1.50–2.40) and enjoyment of sedentary behaviors (OR: 1.41; 95% CI: 1.19–1.68) were related to increased likelihood of being in the high-sedentary-time group. - Boys: Higher scores on self-efficacy (OR: 0.56; 95% CI: 0.44–0.71) was associated with decreased likelihood of being in the high-sedentary-behavior group. - Boys: Higher scores on the cons (OR: 2.15; 95% CI: 1.69–2.73) and enjoyment of sedentary behaviors (OR: 1.49; 95% CI: 1.24–1.80) were associated with increased likelihood of being in the high-sedentary-behavior group. *Multivariate model for girls:* - Included all of the variables that were associated with the outcome from the unadjusted bivariate analyses. - The *R* ^2^ for the main-effects model was 0.25, and the inclusion of the interaction term increased the *R* ^2^ to 0.28. The Hosmer-Lemeshow test indicated that the fit of the model was good ( *P* = .25). *Multivariate model for boys:* - Included age, BMI percentile, cons, and self-efficacy as significant correlates of sedentary time - The final model's *R* ^2^ was 0.22, and the fit of the model was good ( *P* = .35).
Prapavessis, Gaston, & DeJesus, 2015	Convenience sample - N = 372 (283 females, 88 males, one undisclosed) - Adults, between 18 and 64 years of age - Ontario, Canada	Cross-sectional	Theoretical Model: Theory of Planned Behaviour (TPB) Variables: - Attitude - Subjective norms (SN) - Perceived behavioral control (PBC) - Intention with respect to time spent being sedentary	Self-report: Sedentary Behavior Questionnaire (SBQ) - 12-item modified version - Assessed participants' duration of time spent per day in various forms of sedentary pursuits for weekdays and weekends separately. The modified SBQ included both volitional and non-volitional activities.	Single assessment	*TPB variables correlated with sedentary behavior:* - Intention was correlated with attitude (0–4) in only one model, but was related to attitude (half) and attitude (12–16) in three models. Subjective norms were associated with intention in four of the five models and PBC showed an association only in one model. - For behavior, intention emerged as a significant correlate in all five models. Behavior was related with attitude (0–4) in one model, attitude (half) in three models, and attitude (12–16) in two models, SN in three models and PBC in a single model. *Variables predicting sedentary behavior:* - For intention, attitude (half) significantly predicted intention only in Model 5 (weekend leisure/recreation), SN was a significant contributor in three of the five models, and PBC was a significant predictor only in Model 2 (weekday work/school). The percent of variance explained ranged from 9% in Model 3 (weekday leisure/recreation) to 58% inModel 4 (weekend work/school). - For behavior, intention alone significantly predicted behavior in all five models and explained between 2% (Model 3 - weekday leisure/recreation) and 36% (Model 2 - weekday work/school) of the variance. The addition of TPB variables in Step 2 explained an additional 3–11% of the variance in behavior. Attitudes significantly predicted behavior only in Model 2 (weekday work/school) and Model 3 (weekday leisure/recreation). SN significantly predicted behavior in Models 2 (weekday work/school) and 4 (weekend work/school); and PBC significantly predicted behavior only in Model 2 (weekday work/school). Overall, the models explained between 8 and 43% of the variance in behavior.
Quartiroli & Maeda, 2014	Covenience sample - N = 875 - US undergraduat e college students - Wisconsin, USA	Cross-sectional	Theoretical Model: Self-determination theory Variables: - Basic psychological needs in exercise (i.e., perceived competence, autonomy, and relatedness) - Behavioral regulation in exercise (i.e., intrinsic regulation, identified regulation, introjected regulation, external regulation, amotivation) - Relative autonomy index (i.e., degree of self-determination)	Self-report: physical activity and sedentary behavior - International Physical Activity Questionnaire - Self-administered 7-day recall questionnaire - Includes seven items; six measures three levels of physical activity (light, moderate, and vigorous) and one item assesses average daily sitting time as a measure of sedentary behavior.	Single assessment	- Intrinsic regulation ( *r* = –.111, *p* < .001), identified regulation ( *r* = –.074, *p* < .05), autonomy ( *r* = –.092, *p* < .01), competence ( *r* = –.132, *p* < .001), and relatedness ( *r* = –.110, *p* < .001) were all negatively related to sedentary behavior but the correlations were weak. - Although the SDT variables were able to predict some of the variance of sedentary behavior (ρ = −.074 to −.132), the correlations were consistently stronger for predicting MVPA (ρ = .114 to .305), MET min/wk (ρ = .095 to .250), guidelines met (ρ = .114 to .291), and PA guidelines (ρ = .111 to .288). - Psychological needs and behavioral regulation variables together were able to explain 2.8% of the variance of square root transformed sedentary behavior time, *F*(8,866)=3.14, *p* = .002, *R* ^2^ = .028, 90% CI[.006, .040].
Rhodes & Dean, 2009	Random sample - N = 380 - Two samples: Community adult sample (n = 206) and an undergraduat e student sample (n = 174) - Community sample (i.e., adults living in a metropolitan district) drawn from a random sample of residents 18–94 years old; Faculty of Education undergraduat e students volunteered during their certified teacher preparation courses. - Victoria, BC, Canada	Cross-sectional (Community sample) Prospective design (Undergraduat e sample)	Theoretical Model: Theory of Planned Behaviour (TPB) Variables: - Attitude - Subjective norms (SN) - Perceived behavioral control (PBC) - Intention with respect to sedentary leisure behavior	Self-report: - Four sedentary leisure behaviors (television viewing, reading/music, sedentary socializing, and computer use) measured by instrumentati on validated by Salmon et al. (2003) - 1-week recallmeasure (i.e., time spent in each sedentary behaviors in the previous week and weekend) - Average frequency and average duration separated by weekday and weekend	Single assessment (Community sample) Two-week design (Undergradua te sample) - Baseline: TPB variables, self-reported sedentary behavior - Two weeks later: self-reported sedentary behavior	- Results were quite similar across community and undergraduate samples *TPB variables correlated with sedentary behavior:* - For television viewing and computer-use, attitude ( *r* = .37 to .58) and intention ( *r* = .25 to .61) correlated with behavior ( *p* < .01), while perceived behavioral control did not across both samples. Subjective norm correlated with behavior for the community sample ( *r* = .22 to .35; *p* < .01) but not the undergraduate sample. - Intention correlated with behavior for both reading/music ( *r* = .28 to .25) and socializing ( *r* = .31 to .30), but only attitude-reading/music ( *r* = .25), attitude-socializing ( *r* = .29), and subjective norm-socializing ( *r* = .23) relationships were identified for the community sample ( *p* < .01). *Variables predicting sedentary behavior:* TV viewing: - Community sample: attitude ( *β* = .55) and subjective norm ( *β* = .18) predicted intention, *F*(3, 191) = 51.53, *p* < .01, explaining 45% of its variance. Intention ( *β* = .41) was associated with behavior, *F*(1, 181) = 35.78, *p* < .01, and shared 18% of its variance. - Undergraduates: attitude ( *β* = .48) and perceived behavioral control ( *β* = .22) predicted intention, *F*(3, 169) = 38.16; *p* < .01, explaining 40% of its variance. Inturn, intention ( *β* = .41) predicted behavior, *F*(1, 164) = 33.29, *p* < .01, and explained 18% of its variance. Computer use: - Attitude (community sample *β* = .69; undergraduate sample *β* = .54) predicted intention across both community, *F*(3, 180) = 74.57, *p* < .01, R ^2^ = .55 and undergraduate *F*(3, 168) = 45.54, *p* < .01, R ^2^ = .45, samples. - Intention predicted behavior for the community, *F*(1, 170) = 96.15, *p* < .01, R ^2^ = .36 and undergraduate, *F*(1, 163) = 10.63, *p* < .01, R ^2^ = .06, samples. - Attitude also added additional variance as an independent predictor of behavior across both community, **Δ** *F*(3, 167) = 4.07, *p* < .01, R ^2^ change = .04 and undergraduate, **Δ** *F*(3,160) = 6.04, *p* < .01, R ^2^ change = .10, samples. Reading/music: - Attitude (community sample *β* = .41; undergraduate sample *β* = .23) predicted intention in the community, *F*(3, 181) = 45.66, *p* < .01, R ^2^ = .42 and undergraduate, *F*(3, 169) = 8.59, *p* < .01, R ^2^ = .13 samples, though perceived behavioral control ( *β* = .24) was also a predictor in the community sample. - Intention predicted behavior for both community, *F*(1,178) = 15.56, *p* < .01, R ^2^ = .08 and undergraduate, *F*(1, 162) = 10.47, *p* < .01, R ^2^ = .06, samples. Socializing: - Attitude predicted intention across both models (community sample *β* = .47; undergraduate sample *β* = .38), while subjective norm ( *β* = .29) was a predictor in the community sample and perceived behavioral control ( *β* = .43) was a predictor in the undergraduate sample. Overall, both the community sample, *F*(3, 189) = 108.06, *p* < .01, R ^2^ = .63 and the undergraduate sample, *F*(3, 169) = 34.55, *p* < .01, R ^2^ = .38, were significant. - Intention also predicted behavior across both community, *F*(1, 177) = 17.56, *p* < .01, R ^2^ = .09 and undergraduate, *F*(1, 163) = 17.00, *p* < .01, R ^2^ = .09, samples.
Salmon, Owen, Crawford, et al., 2003	Random sample - N = 1,332 - Population-based mail survey of Australian adults - Australia	Cross-sectional	Psychological, theory-driven: Behavioral choice theory (BCT) - Incorporates both individual level and environmental influences Variables: - Barriers to physical activity (environmental, personal) - Enjoyment of physical activities - Enjoyment of sedentary behaviors - Preference for physical activity or sedentary behavior	Self-report: Leisure-time sedentary behavior: - 1-week recall measure (time spent in nine sedentary behaviors in the previous Monday– Friday and weekend [Saturday and Sunday]) - Television viewing was dichotomized as low (< 14 hr/week) and high (> 14 hr/week); reading was dichotomized as low (< 5 hr/week) and high (> 5 hr/week); and sitting socializing was dichotomized as low (< 8 hr/week) and high (> 8 hr/week). Leisure-time physical activity: - 1-week leisure-time physical activity recall measure - Frequency and duration of participation in walking, moderate-intensity activity, vigorous activity, and total leisure-time activity.	Single assessment	*Associations of Barriers, Enjoyment, and Preferences with Sedentary Behavior:* - Multivariate logistic regression analyses were performed to predict the likelihood of being a high television viewer (> 14 hr/week), the likelihood of reading more than 5 hr/week, the likelihood of sitting and socializing more than 8 hr/week, and the likelihood of spending more than 36 hr/week in a total of nine leisure-time sedentary pursuits. *Variables predicting high participation in television viewing:* - Multiple linear regression explained 14.5% of the variance in television viewing, *F*(22, 1251) = 11.0, *p* < .01, with enjoyment of television viewing explaining the greatest proportion of variance ( *R ^2^* = 10.2, *β* = 0.3, *p* < .01); then physical activity barriers such as the weather ( *R ^2^* = 1.1, *β* = 0.10, *p* < .01), work commitments ( *R ^2^* = 0.9, *β* = –0.11, *p* < .01), feeling tired ( *R ^2^* = 0.5, *β* = 0.06, *p* < .05), and cost ( *R ^2^* = 0.3, *β* = 0.06, *p* < .05); and preference for vigorous physical activity ( *R ^2^* = 0.3, *β* = –0.06, *p* < .05). *Variables predicting reading more than 5 hr/week:* - Multiple linear regression explained 17.2% of the variance in reading, *F*(22, 1251) = 13.1, *p* < .01, with enjoyment of reading explaining the greatest pro-portion of variance ( *R ^2^* = 11.1, *β* = 0.34, *p* < .01); physical activity barriers such as family commitments ( *R ^2^* = 1.2, *β* = –0.09, *p* < .01), the weather ( *R ^2^* = 0.6, *β* = 0.07, *p* < .01), work commitments ( *R ^2^* = 0.6, *β* = –0.09, *p* < .01), and lack of safety ( *R ^2^* = 0.3, *β* = 0.06, *p* < .05). *Variables predicting sitting and socializing more than 8 hr/week:* - Multiple linear regression explained 15.8% of the variance in sitting socializing, *F*(22, 1251) = 11.4, *p* < .01, with enjoyment of socializing explaining the greatest proportion of variance ( *R ^2^* =9.1, *β* = 0.23, *p* < .01); then physical activity barriers such as family commitments ( *R ^2^* = 0.6, *β* = –0.08, *p* < .01), pollution ( *R ^2^* = 0.4, *β* = 0.07, *p* < .01), and work commitments ( *R ^2^* = 0.3, *β* = –0.07, *p* < .05); and preference for sedentary behavior ( *R ^2^* = 0.3, *β* = 0.06, *p* < .05). *Variables predicting high participation in leisure-time sedentary behavior:* - The amount of variance that was explained for total sedentary behavior was 13.3%, *F*(22, 1251) = 9.2, *p* < .01, with enjoyment of sedentary behavior explaining the greatest proportion of variance ( *R ^2^* = 4.9, *β* = 0.20, *p* < .01); then physical activity barriers such as the weather ( *R ^2^* = 1.4, *β* = 0.10, *p* < .01), family commitments ( *R ^2^* = 1.5, *β* = –0.12, *p* < .01), work commitments ( *R ^2^* = 0.7, *β* = –0.14, *p* < .01), feeling tired ( *R ^2^* = 1.0, *β* = 0.09, *p* < .01), and pollution ( *R ^2^* = 0.5, *β* = 0.08, *p* < .01); age ( *R ^2^* = 0.5, *β* = –0.07, *p* < .05); and preference for sedentary behavior ( *R ^2^* = 0.4, *β* = 0.13, *p* < .01), enjoyment of structured physical activity ( *R ^2^* = 0.4, *β* = 0.09, *p* < .01), and preference for moderate physical activity ( *R ^2^* = 0.3, *β* = 0.08, *p* < .05).
Van Dyck, Cardon, Deforche, et al., 2011	Random sample - N = 419 - Adults - Ghent, Belgium	Cross-sectional	Theory-driven: Ecological model Variables: - Socio-demographic (gender; age; educational attainment [primary, secondary, tertiary education]; employment status [employed, not employed/retired]; and body mass index) - Sedentary-specific home-environmental (number of TVs and computers in home, size of largest TV set) - Sedentary-specific psychosocial (Pros and cons of reducing screen time, self-efficacy about reducing screen time, and social norm from family and friends)	Self-report: Domestic screen time - Self-reported TV viewing time (min/day) and leisure-time internet use at home (min/day) - ‘Usual week’ assessed	Single assessment	*Bivariate correlations of psychosocial factors with TV viewing:* - Pros reducing TV viewing ( *r* = –0.31, *p* < .001) - Cons reducing TV viewing ( *r* = 0.47, *p* < .001) - Family social norm TV viewing ( *r* = 0.34, *p* < .001) - Friends social norm TV viewing ( *r* = 0.35, *p* < .001) - Self-efficacy reducing TV viewing ( *r* = –0.49, *p* < .001) *Bivariate correlations of psychosocial factors with internet use:* - Pros reducing internet use ( *r* = –0.16, *p* < .01) - Cons reducing internet use ( *r* = 0.31, *p* < .001) - Family social norm internet use ( *r* = 0.40, *p* < .001) - Friends social norm internet use ( *r* = 0.26, *p* < .001) - Self-efficacy reducing internet use ( *r* = –0.47, *p* < .001) *Associations of psychosocial variables with TV viewing time:* - For the psychosocial variables, perceiving more cons was associated with more TV viewing time ( *β* = 0.155, *p* = 0.014) while more pros ( *β* = –0.177, *p* < 0.001) and higher self-efficacy about reducing TV viewing time were related to less TV viewing time ( *β* = –0.241, *p* < 0.001). *Associations of psychosocial variables with leisure-time internet use:* - Concerning the psychosocial factors, perception of higher social norm from family towards Internet use ( *β* = 0.161, *p* = 0.011) and more cons ( *β* = 0.187, *p* = 0.002) were related to more leisure-timeInternet use. Moreover, more pros ( *β* = –0.116, *p* = 0.009) and higher self-efficacy about reducing leisure-time Internet use were associated with less Internet use ( *β* = –0.285, *p* < 0.001).
Wallmann-Sperlich, Bucksch, Schneider, et al., 2014	Representative sample - N = 1515; 747 men; 43.5 ± 11.0 years - Working German adults - Germany	Cross-sectional	Non-theory driven: Socio-demographic, behavioural and cognitive correlates Variables: Socio-demographic: - age, education level, income levelBehavioural: - work-related PA, travel-related PA, leisure-related PA as well as sitting time during transport, during TV watching, during leisure computer use and during leisure time Cognitive: - Health-related beliefs about sitting time	Self-report: Marshall Sitting Questionnaire - Five items were used to assess time spent in specific sitting pursuits (hours and minutes) each day in five domains on weekdays and weekend days. - Dependent variable was sitting time during work on weekdays. All sitting time measures other than work-related on weekdays was considered independent variables. Global Physical Activity Questionnaire (GPAQ) - Used to assess PA	Single assessment	*Correlates of work-related sitting time:* - The only association with cognitive correlates was found in men for the belief ‘Sitting for long periods does not matter to me’ ( *β* = .10) expressing a more positive attitude towards sitting with increasing sitting durations. *Variables predicting work-related sitting time:* - In model 4, for men, the belief ‘Sitting for long periods does not matter to me’ (recoded) ( *β* = .10) was positively correlated with work-related sitting time, reflecting more positive attitudes towards sitting with increasing sitting durations. - For women, for the cognitive variables, no associations were found.
Wong, Gaston, DeJesus, et al., 2016	Convenience sample - N = 596 - Undergraduat e university students, aged 18-35 years - Ontario, Canada	Prospective study - After completing socio-demographics and the PMT items, participants randomized to complete general or leisure GI and II. Based on model assignment, they completed either the general or leisure SB questionnaire one week later.	Theoretical Model: Protection Motivation Theory (PMT) Sedentary-derived PMT variables: - Threat appraisals: perceived vulnerability (PV), perceived severity (PS) - Coping appraisals: response efficacy (RE), scheduling self-efficacy (SE) - SE subscales: three psychological (productive, focused, tired), and two situational (studying, leisure) - Intention: goal intention (GI), implementation intention (II)	Self-report: Sedentary Behavior Questionnaire (SBQ) - 12-item modified version - Measured the quantity of time spent sitting on a typical day over the previous week - Seven items assessed leisure-specific, volitional sedentary activities Exercise behavior: Leisure Score Index (LSI) of the Leisure Time Exercise Questionnaire - Four-item assessment that measures intensity and frequency of physical activity	7-day period - Baseline: PV, PS, RE, SE, II, GI, LSI - One week later: modified SBQ - PMT cognitions were assessed prior to sedentary behavior	*PMT variables correlated with sedentary behavior:* - In the general model, scheduling SE productive/focused ( *r* = –.13, *p* < .05) and scheduling SE studying in library/Wi-Fi area ( *r* = –.14, *p* < .05) were significantly related to sedentary behavior. - In the leisure model, PV ( *r* = .12, *p* < .05), scheduling SE TV/video games/computer ( *r* = –.13, *p* < .05), scheduling SE studying in library/Wi-Fi ( *r* = –.11, *p* < .05) and goal intention ( *r* = .20, *p* < .05) were significantly related tosedentary behavior. *Variables predicting sedentary behavior:* - For goal intention, 5% and 1% of the variance was explained in the general and leisure model, respectively. RE and scheduling SE studying at home were significant contributors for the general model only. - For implementation intention, 10% and 16% of the variance was explained in the general and leisure model, respectively. In the general model, PV, RE, and scheduling SE productive/focused were significant contributors. For the leisure model, PV, RE, and scheduling SE studying at homewere significant contributors. - For sedentary behavior, 3% and 1% of the variance was explained in the general and leisure model, respectively. Goal intention was a significant contributor in the leisure model only.
